# *Pax9* is required for cardiovascular development and interacts with *Tbx1* in the pharyngeal endoderm to control 4th pharyngeal arch artery morphogenesis

**DOI:** 10.1242/dev.177618

**Published:** 2019-09-15

**Authors:** Helen M. Phillips, Catherine A. Stothard, Wasay M. Shaikh Qureshi, Anastasia I. Kousa, J. Alberto Briones-Leon, Ramada R. Khasawneh, Chloe O'Loughlin, Rachel Sanders, Silvia Mazzotta, Rebecca Dodds, Kerstin Seidel, Timothy Bates, Mitsushiro Nakatomi, Simon J. Cockell, Jürgen E. Schneider, Timothy J. Mohun, René Maehr, Ralf Kist, Heiko Peters, Simon D. Bamforth

**Affiliations:** 1 Institute of Genetic Medicine, Newcastle University, Newcastle-upon-Tyne NE1 3BZ, UK; 2 Memorial Sloan Kettering Cancer Center, New York, NY 10065, USA; 3 School of Dental Sciences, Newcastle University, Newcastle-upon-Tyne NE2 4BW, UK; 4 Bioinformatics Support Unit, Newcastle University, Newcastle-upon-Tyne NE2 4HH, UK; 5 Biomedical Imaging, University of Leeds, Leeds LS2 9JT, UK; 6 The Francis Crick Institute, London NW1 1AT, UK; 7 Diabetes Center of Excellence, University of Massachusetts Medical School, Worcester, MA 01605, USA

**Keywords:** Pharyngeal endoderm, Arch artery development, *Tbx1*, *Pax9*, Neural crest, 22q11 deletion syndrome

## Abstract

Developmental defects affecting the heart and aortic arch arteries are a significant phenotype observed in individuals with 22q11 deletion syndrome and are caused by a microdeletion on chromosome 22q11. *TBX1*, one of the deleted genes, is expressed throughout the pharyngeal arches and is considered a key gene, when mutated, for the arch artery defects. *Pax9* is expressed in the pharyngeal endoderm and is downregulated in *Tbx1* mutant mice. We show here that *Pax9*-deficient mice are born with complex cardiovascular malformations that affect the outflow tract and aortic arch arteries with failure of the 3rd and 4th pharyngeal arch arteries to form correctly. Transcriptome analysis indicated that *Pax9* and *Tbx1* may function together, and mice double heterozygous for *Tbx1*/*Pax9* presented with a significantly increased incidence of interrupted aortic arch when compared with *Tbx1* heterozygous mice. Using a novel *Pax9Cre* allele, we demonstrated that the site of this *Tbx1-Pax9* genetic interaction is the pharyngeal endoderm, therefore revealing that a *Tbx1*-*Pax9*-controlled signalling mechanism emanating from the pharyngeal endoderm is required for crucial tissue interactions during normal morphogenesis of the pharyngeal arch artery system.

## Abstract (precis)

**Summary:** A strong genetic interaction between *Tbx1* and *Pax9* that leads to 4th PAA-derived defects in double heterozygous mice is cell-autonomous within the pharyngeal endoderm.

## INTRODUCTION

Conotruncal heart malformations, which affect the outflow tract and aortic arch arteries, occur in 30% of all cases of congenital heart defects (Thom et al., 2006). Approximately 20% of foetuses identified with conotruncal defects by ultrasound are diagnosed with 22q11 deletion syndrome (22q11DS) (Boudjemline et al., 2001), the most common microdeletion syndrome with an incidence of 1:4000 live births (Scambler, 2000). Patients typically have a 3 Mb deletion on chromosome 22 that encompasses 45 protein-coding genes (Morrow et al., 2018) and ∼80% of patients present with some form of congenital cardiovascular defect (Momma, 2010), of which one of the most common observed is interruption of the aortic arch (IAA) (Unolt et al., 2018). Although clinically rare in the general population, ∼50% of all cases of IAA occur in 22q11DS patients (Boudjemline et al., 2001; Lewin et al., 1997; Van Mierop and Kutsche, 1986). IAA is a consequence of the left 4th pharyngeal arch artery (PAA) failing to form correctly during embryonic development. There are five pairs of PAAs that arise within a series of repeated protuberances on either side of the developing pharynx known as the pharyngeal arches. These arches consist of paraxial mesoderm- and neural crest cell (NCC)-derived mesenchyme, and are externally enclosed by ectoderm and internally lined by endoderm (Graham, 2003). The initially bilaterally symmetrical PAAs develop sequentially in a cranial to caudal sequence during embryogenesis but undergo a complex remodelling process, which is conserved in mammals, to form the asymmetrical mature aortic arch configuration (Bamforth et al., 2013; Hiruma et al., 2002). Of the genes deleted in 22q11DS, hemizygosity of *TBX1* is considered to be the cause of the cardiovascular defects, with point mutations in *TBX1* also identified in individuals with 22q11DS-like phenotypes (Yagi et al., 2003; Zweier et al., 2007). Tbx1 is expressed throughout the pharyngeal arches within the ectoderm, endoderm and mesoderm cells, and is crucial for pharyngeal arch and PAA development (Jerome and Papaioannou, 2001; Lindsay et al., 2001; Merscher et al., 2001). Complete loss of *Tbx1* from the mouse results in a failure of the pharyngeal arches to form correctly, resulting in hypoplasia of the second arch and aplasia of the remaining caudal arches, leading to an absent thymus and common arterial trunk (Jerome and Papaioannou, 2001; Merscher et al., 2001). In contrast, heterozygous deletion of *Tbx1* predominantly affects development of the 4th PAA resulting in IAA in a minority of mice with aberrant right-subclavian artery (A-RSA) observed more frequently (Lindsay et al., 2001). *Tbx1* heterozygotes may, however, also present with VSD, overriding aorta, double aortic arch and an abnormal thymus (Merscher et al., 2001; Zhang and Baldini, 2008). Although almost all 22q11DS patients are hemizygous for the deletion, the phenotypic spectrum is highly variable (Unolt et al., 2018); thus, it has been proposed that genes outside of the deleted region may impact on the clinical phenotype (Guo et al., 2011b). Several genes have been identified as potential modifiers of 22q11DS, as determined by genetic interaction studies and transcriptome analyses in *Tbx1* mutant mice (Aggarwal and Morrow, 2008; Ivins et al., 2005; Liao et al., 2008; Papangeli and Scambler, 2013). One such candidate gene is *Pax9* the expression of which in the pharyngeal endoderm was found to be significantly reduced in *Tbx1*-deficient embryos (Ivins et al., 2005; Liao et al., 2008). *Pax9* belongs to a family of Pax genes encoding transcription factors involved in various cellular roles during embryonic development (Chi and Epstein, 2002; Mansouri et al., 1996) and is specifically expressed in the endoderm of all four pharyngeal pouches of the mouse by E9.5 (Neubüser et al., 1995). At later stages in mouse development, Pax9 is expressed in the oesophagus, somites and limbs, and within the neural crest contributing to craniofacial development (Peters et al., 1998). Embryos deficient for *Pax9* exhibit craniofacial defects, including a cleft secondary palate as well as absent teeth, skeletal defects and lack derivatives of the 3rd and 4th pharyngeal pouches, i.e. the thymus, parathyroids and ultimobranchial bodies (Peters et al., 1998), yet the role of Pax9 in cardiovascular development has not been determined.

In this study, we have investigated cardiovascular development in *Pax9*-deficient mice, and show that loss of *Pax9* leads to complex cardiovascular defects affecting the outflow tract and aortic arch arteries. We also uncover a strong genetic interaction between *Tbx1* and *Pax9* that leads to 4th PAA-derived defects in double heterozygous mice; this interaction is cell-autonomous within the pharyngeal endoderm.

## RESULTS

### *Pax9*-deficient embryos have cardiovascular defects

**Fig. 1.**
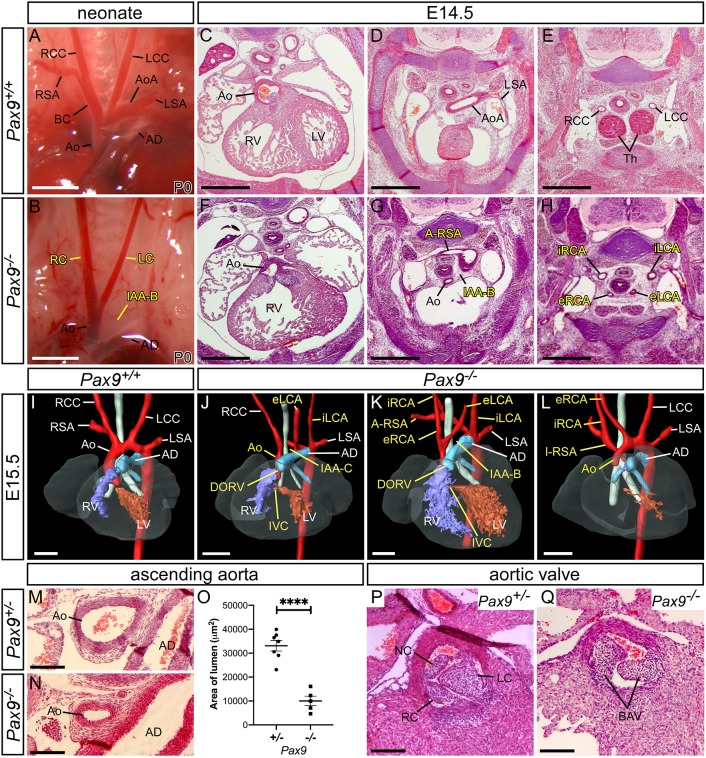
**Loss of *Pax9* results in cardiovascular developmental defects.** (A) Arch arteries of control neonates were normal (*n*=8). (B) *Pax9^–/–^* neonates (*n*=5) displayed IAA-B, absent right subclavian artery (presumed retro-oesophageal), and atypical right and left carotid arteries (LC and RC). Scale bars: 1 mm. (C-E) *Pax9^+/+^* embryos at E14.5 show normal outflow tract, arch arteries and thymus. (F-H) In *Pax9^–/–^*embryos (*n*=4), the aorta (Ao) arises aberrantly from the right ventricle producing a double outlet right ventricle (DORV; F). Hypoplastic aorta, IAA-B and an A-RSA are present (G). More cranially, the thymus is absent and aberrant internal and external carotid arteries (iRCA, eRCA, iLCA, eLCA) are seen (H). (I-L) 3D reconstructions of E15.5 hearts from MRI datasets. (I) *Pax9^+/+^* embryo with normal aortic arch arteries. (J-L) In *Pax9^–/–^*embryos (*n*=15), defects seen are DORV with interventricular communication (IVC), IAA (type B or C), A-RSA (retro-esophageal in J,K and isolated in L) and aberrant carotid arteries. Scale bars: 500 μm. (M-O) The ascending aorta is significantly smaller in *Pax9^–/–^* embryos (*n*=5) compared with *Pax9^+/–^* control embryos (*n*=7). Data are mean±s.e.m. ****P<0.0001 (two-tailed unpaired *t*-test). (P) *Pax9^+/–^* control embryos have three normal aortic valve leaflets (*n*=6): the right (RC), left (LC) and non-coronary (NC). (Q) *Pax9^–/–^* embryos (*n*=5) have bicuspid aortic valve (BAV). Scale bar: 100 μm. AoA, aortic arch; AD, arterial duct; BC, brachiocephalic; dAo, dorsal aorta; LCC, left common carotid artery; LSA, left subclavian artery; LV, left ventricle; RCC, right common carotid artery; RSA, right subclavian artery; RV, right ventricle.

Mice lacking *Pax9* die perinatally and exhibit craniofacial, pharyngeal gland and skeletal defects (Peters et al., 1998). As the cause of death was unclear, we carefully re-analysed *Pax9^–/–^* neonates from a *Pax9^+/–^* intercross and found that all *Pax9^–/–^* neonates had a patterning defect of their aortic arch arteries that included an IAA with A-RSA (Fig. 1B; Table 1). To investigate these defects in more detail, we processed *Pax9^–/–^* embryos for histology and magnetic resonance imaging (MRI), and found that *Pax9^–/–^* embryos presented with a wide range of cardiovascular abnormalities (Fig. 1C-Q; Table 1). These defects included double outlet right ventricle with interventricular communication (Fig. 1F,J,K), IAA and A-RSA (Fig. 1G,K). The common carotid arteries were also frequently absent, resulting in unilateral or bilateral internal and external carotids arising directly from the dorsal aorta and the A-RSA (Fig. 1H,J-L). *Pax9^–/–^* embryos also had a hypoplastic aorta (Fig. 1N-O) and bicuspid aortic valves (Fig. 1Q).

**Table 1.**
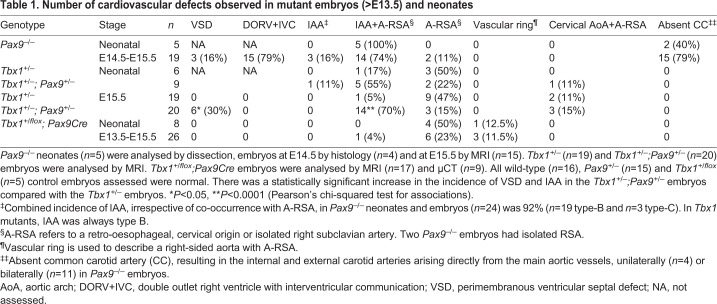
**Number of** **cardiovascular defects observed in mutant embryos (>E13.5) and neonates**

**Fig. 2.**
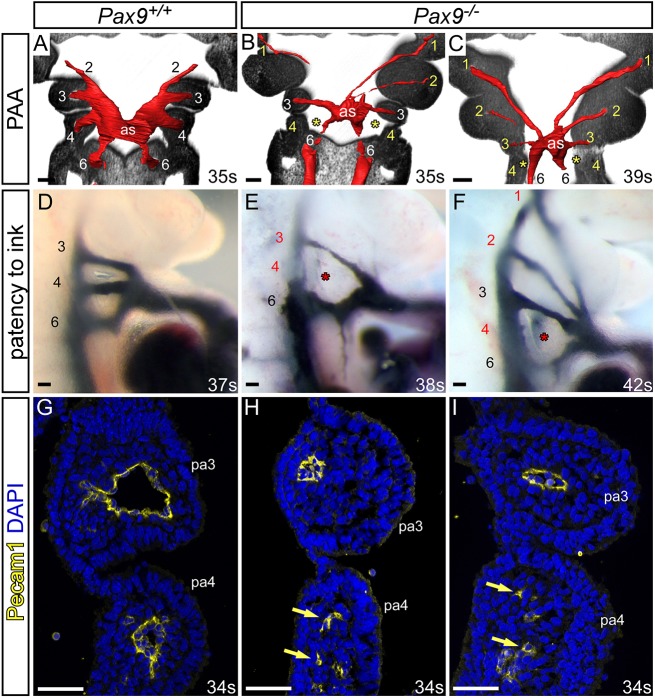
***Pax9* loss causes aberrations in pharyngeal arch artery formation.** (A-C) Coronal views of control (*Pax9^+/+^*) and mutant (*Pax9^–/–^*) embryos at E10.5 (30-40 s), examined by high-resolution episcopic microscopy. (A) In *Pax9^+/+^* control embryos (*n*=5), the 3rd, 4th and 6th PAAs are of equal size and bilaterally symmetrical. (B,C) In *Pax9^–/–^* embryos (*n*=4), the 4th PAAs were bilaterally absent (asterisk), the 3rd PAAs and aortic sac (as) were hypoplastic, and the 1st and/or 2nd PAAs abnormally persisted. (D-F) Intracardiac ink injection into E10.5-E11.0 (31-42 s) embryos. (D) In control embryos (*n*=12), PAAs 3-6 are patent to ink, are of equivalent diameter and are bilaterally symmetrical. (E,F) In *Pax9^–/–^* embryos (*n*=12), the 3rd PAAs are hypoplastic and the 4th PAAs are non-patent to ink (asterisk). (F) The 1st and 2nd PAAs are persisting anomalously. (G-I) Immunostaining using anti-Pecam1 antibody at E10.5 (31-36 s). (G) Control embryos (*n*=3) have a ring of Pecam1-positive endothelium lining the 3rd and 4th PAAs. (H,I) In *Pax9^–/–^* embryos (*n*=5), the 3rd PAAs are visibly smaller and disorganized endothelial cells are within the 4th pharyngeal arch (pa; arrows). Somite counts (s) are indicated. Scale bars: 100 μm. The somite numbers given in the legend reflect the range analysed for the whole study. The figure contains representative images only.

Developmental defects of the arch arteries occur when the PAAs fail to form or remodel correctly. To investigate the morphogenesis of the PAAs, we performed high-resolution episcopic microscopy (HREM) on E10.5 and E11.5 *Pax9^–/–^* embryos and manually segmented the datasets to create 3D images of the PAA. Embryos were stage-matched by somite counting to exclude developmental delay as a cause of observed defects. Analysis of E10.5 coronal sections revealed that the 3rd and 4th pharyngeal arches of *Pax9^–/–^* embryos were smaller compared with *Pax9^+/+^* controls, as previously reported (Peters et al., 1998), but did show segmentation to individual arches (Fig. 2A-C). At E10.5, the developing PAAs are bilaterally symmetrical, having formed in a cranial to caudal sequence. The 1st and 2nd PAAs have normally regressed by this stage, and the 3rd, 4th and 6th PAAs are present (Bamforth et al., 2013; Hiruma et al., 2002) (Fig. 2A). In *Pax9^–/–^* embryos, the 1st and 2nd PAAs were abnormally persistent, the 3rd PAAs were either absent, hypoplastic or interrupted, and the 4th PAAs were absent (Fig. 2B,C; Table 2). The aortic sac was also hypoplastic in *Pax9^–/–^* embryos compared with the controls (Fig. 2B,C). Intra-cardiac injection of India ink at E10.5-E11.0 showed that, in controls, the caudal PAAs were patent to ink and of equivalent size (Fig. 2D). In *Pax9^–/–^* embryos, however, persisting 1st and 2nd PAAs were patent to ink, and the 3rd PAAs were hypoplastic (Fig. 2J-L) or non-patent to ink, and therefore presumed absent. The majority of the 4th PAAs were bilaterally non-patent to ink (Fig. 2J-L; Table 2). Immunohistochemical staining for Pecam1 showed that the endothelium within the 3rd and 4th pharyngeal arches had formed lumenised PAAs at E10.5 in control embryos (Fig. 2G), but in *Pax9^–/–^* embryos the 3rd PAAs were visibly smaller in size and only isolated endothelial cells were seen within the 4th pharyngeal arch (Fig. 2H,I).

**Table 2.**
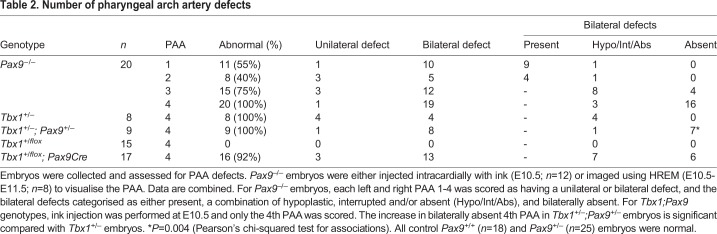
**Number of** **pharyngeal arch artery defects**

**Fig. 3.**
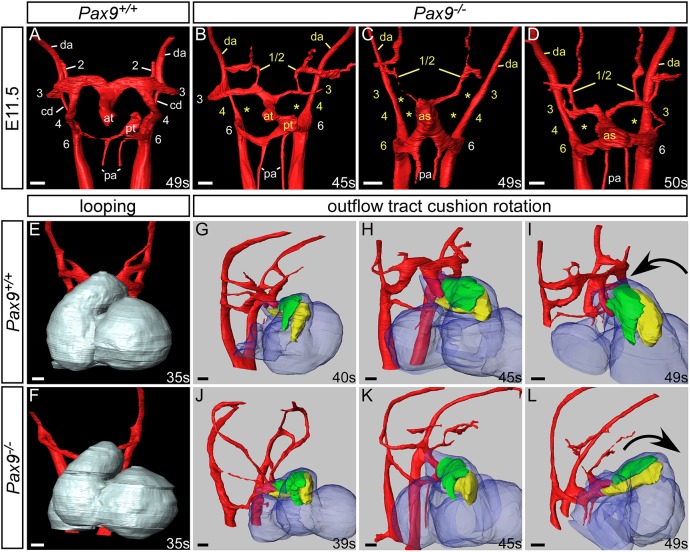
***Pax9^–/–^* embryos display aberrant rotation of the outflow tract and abnormal PAA remodelling.** Image datasets were acquired by high-resolution episcopic microscopy. (A-D) 3D reconstructions of the PAAs at E11.5 (45-50 s). (A) In *Pax9^+/+^* control embryos (*n*=5), the outflow tract is septated, the right 6th PAA has thinned and the carotid duct (cd) has begun to involute. (B-D) In *Pax9^–/–^* embryos (*n*=4), the 4th PAAs are absent and the 3rd PAAs are aberrantly connected or detached from the dorsal aorta (da; asterisks). The carotid duct has failed to involute and septation of the outflow tract is delayed. Persisting 1st or 2nd PAAs are observed coursing anteriorly from the abnormal 3rd PAAs (B,D) or from the aortic sac (as; C). (E,F) Looping of the heart tube at E10.5 was not affected in *Pax9^–/–^* embryos (*n*=4). (G-L) Outflow tract rotation from E10.5 to E11.5 (39-49 s). (G-I) In control embryos, the major outflow tract cushions (parietal in green; septal in yellow) rotated in an anti-clockwise direction, resulting in the cushions being aligned side by side. (J-L) In *Pax9^–/–^* embryos, the cushions rotated in a clockwise direction, resulting in the parietal cushion being anterior to the septal cushion. at, aortic trunk; pa, pulmonary arteries; pt, pulmonary trunk. Somite (s) counts indicated. Scale bars: 100 μm.

During E11.5 of mouse development, the PAA system begins to remodel and becomes asymmetric in appearance (Bamforth et al., 2013; Hiruma et al., 2002). In *Pax9* control embryos at this stage, the right 6th PAAs appeared thinner and the distal outflow tract was septated. The carotid duct, the region of the dorsal aorta between the 3rd and 4th PAAs, had begun to involute, but the 3rd, 4th and 6th PAAs remained connected to the dorsal aortae (Fig. 3A). In *Pax9^–/–^* embryos at E11.5, presumed persisting 1st or 2nd PAAs were observed extending anteriorly from the aortic sac, and the 3rd PAAs were also affected, either unilaterally or bilaterally, being either absent, hypoplastic or interrupted and not connected to the dorsal aortae (Fig. 3B-D). The carotid ducts were maintained, and this, coupled with the abnormal detachment of the 3rd PAAs from the dorsal aorta and the persistence of the 1st or 2nd PAAs, resulted in an absent common carotid artery and the internal and external carotid arteries arising directly from the aortic arch arteries, as observed at E15.5 (Fig. 1). Additionally, the distal outflow tract was unseptated and the 4th PAAs were bilaterally absent (Fig. 3B-D; Table 2).

Looping of the heart tube at E10.5 was not affected in *Pax9^–/–^* embryos (Fig. 3E,F).

In normal mouse development, the outflow tract cushions rotate through an anticlockwise direction (Bajolle et al., 2006), resulting in the major septal and parietal cushions being positioned side by side (Fig. 3G-I). In *Pax9^–/–^* embryos, however, by the end of the E11.5 stage these cushions were aberrantly positioned, with the parietal cushion more anterior to the septal cushion, indicating that a clockwise rotation of the outflow tract cushions had occurred (Fig. 3J-L).

### Pharyngeal endoderm signalling influences neural crest cell differentiation

**Fig. 4.**
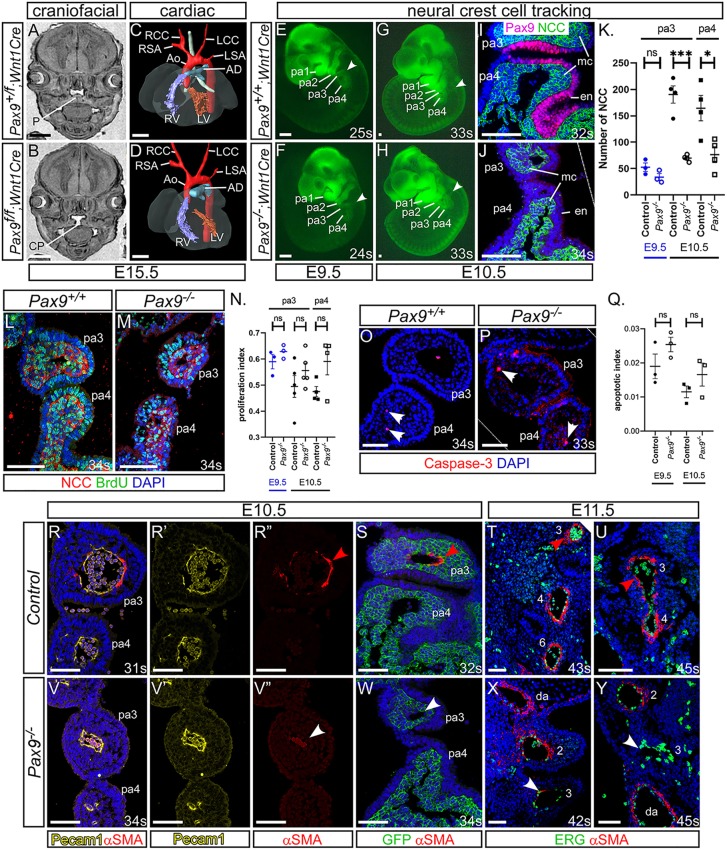
**Failure in smooth muscle cell recruitment causes the 3rd PAAs to collapse.** (A-D) E15.5 embryos with a *Pax9* conditional deletion in neural crest cells (NCCs) were examined by MRI. (A) Control embryos had a normal palate (P). (B) *Pax9^f/f^;Wnt1Cre* mutant embryos (*n*=6) had cleft palate (CP). (C,D) No cardiovascular defects were observed in control or mutant embryos. Ao, aorta; AD, arterial duct; LCC, left common carotid artery; LSA, left subclavian artery; LV, left ventricle; RCC, right common carotid artery; RSA, right subclavian artery; RV, right ventricle. (E-J) NCCs in *Pax9^+/+^* (E,G,I) or *Pax9^–/–^* (F,H,J) embryos were labelled using *Wnt1Cre* and *eYFP* alleles. (E-H) Fluorescence imaging showed no difference in NCC migration into the pharyngeal arches (arrowheads) at E9.5 (*n*≥3, 23-25 s) and E10.5 (*n*≥5, 32-36 s). (I,J) Fluorescent embryos were sectioned coronally and immunostained using anti-GFP and anti-Pax9 antibodies. (K) NCCs were counted from immunostained sections. *P<0.05, ***P<0.001 (two-tailed unpaired *t*-test). (L,M) Embryo sections were immunostained with anti-BrdU and anti-GFP antibodies to detect proliferation in NCCs in *Pax9^+/+^* (L) and *Pax9^–/–^* (M) embryos. (N) No significant difference in the rate of proliferation was found between control and *Pax9^–/–^* NCCs in the caudal arches at E9.5 (*n*=3, 23-28 s) or E10.5 (*n*≥4, 31-39 s). (O,P) Embryo sections immunostained using an anti-caspase 3 antibody to detect apoptosis in the caudal pharyngeal arches of *Pax9^+/+^* (N,O) and *Pax9^–/–^* (P) embryos. (Q) No significant difference in the rate of apoptosis was found between control and *Pax9^–/–^* embryos at E9.5 (*n*=3, 24-28 s) or E10.5 (*n*=3, 30-35 s). Two-tailed unpaired *t*-test. (R-Y) Embryo sections were immunostained using anti-αSMA antibody for smooth muscle (R,R″-U,V,V″-Y,S,W), anti-Pecam1 (R,R′,V,V′) or -ERG (T,U,X,Y) antibodies for endothelium, or anti-GFP to label NCCs in *Wnt1Cre;eYFP* embryos (S,W). In all control embryos, SMC surrounded the 3rd PAAs [E10.5, *n*=7, 31-36 s (R,S); and E11.5, *n*=3, 43-45 s (T,U); red arrowhead]. In *Pax9^–/–^* embryos, the 3rd PAAs had limited recruitment of SMCs [E10.5, *n*=6; 32-35 s (V,W); and E11.5, *n*=3, 42-45 s (X,Y); white arrowheads]. da, dorsal aorta; en, endoderm; mc, mesenchyme; ns, not significant; pa, pharyngeal arch. Somite counts (s) are indicated. Scale bars: 500 μm in A-D; 100 μm in E-J,L,M,O,P; 50 µm in R-Y. The somite numbers given in the legend reflect the range analysed for the whole study. The figure contains representative images only.

During normal embryogenesis, a subset of NCCs, the cardiac neural crest cells, migrate into the caudal pharyngeal arches to provide support to the developing PAAs and also contribute to outflow tract septation (Hutson and Kirby, 2003). *Pax9* is predominantly expressed in the pharyngeal endoderm (Fig. S1), but is also known to be expressed within the NCC and contributes to craniofacial development (Kist et al., 2007), although expression in cardiac NCC has not been described. We therefore looked to see whether a cell-autonomous loss of *Pax9* from NCC resulted in cardiovascular defects. Conditional deletion of *Pax9* using *Wnt1Cre* transgenic mice (Danielian et al., 1998), and the *Pax9^flox^* allele (Kist et al., 2007), however, did not result in cardiovascular abnormalities, although cleft palate was observed as expected (Fig. 4A-D). We next looked to see whether the migration of NCCs was affected in global *Pax9^–/–^* embryos using *Wnt1Cre* and *eYFP* (Srinivas et al., 2001) reporter mice to create embryos where the NCC could be lineage traced. Live fluorescence imaging of *Pax9^+/+^;Wnt1Cre;eYFP* embryos at E9.5 and E10.5 revealed NCCs present in the head and dorsal root ganglia, as well as migrating towards and filling the pharyngeal arches (Fig. 4E,G). A similar pattern was observed in *Pax9^–/–^;Wnt1Cre;eYFP* embryos (Fig. 4F,H). Counting of NCCs revealed no significant change in their number in the 3rd pharyngeal arch at E9.5, but a significant reduction was found in the 3rd and 4th arches at E10.5 (Fig. 4K). Immunostaining for BrdU incorporation specifically in NCCs showed no significant difference in NCC proliferation in the caudal pharyngeal arches (Fig. 4L-N), and very little apoptosis was seen in both control and *Pax9^–/–^* embryos (Fig. 4O-Q). These observations indicated that the migration of NCCs per se to the pharyngeal arches was not affected in *Pax9^–/–^* embryos. Given that NCCs differentiate into the smooth muscle cells (SMCs) that support the remodelling PAAs (Waldo et al., 1996), we looked to see whether SMC recruitment to the 3rd PAAs was affected in *Pax9^–/–^* embryos. Sections from E10.5 and E11.5 embryos were immunostained using an anti-alpha smooth muscle actin antibody to visualise SMCs around the PAAs. Evidence of SMC recruitment to the 3rd PAAs was seen in each control embryo examined (Fig. 4R-U); however, very little SMC recruitment to the 3rd PAAs in *Pax9^–/–^* embryo sections was observed (Fig. 4V-Y). These results indicate that the 3rd PAAs in *Pax9^–/–^* embryos form but collapse from a failure of SMC recruitment.

### *Tbx1* and *Pax9* interact in 4th PAA formation

**Fig. 5.**
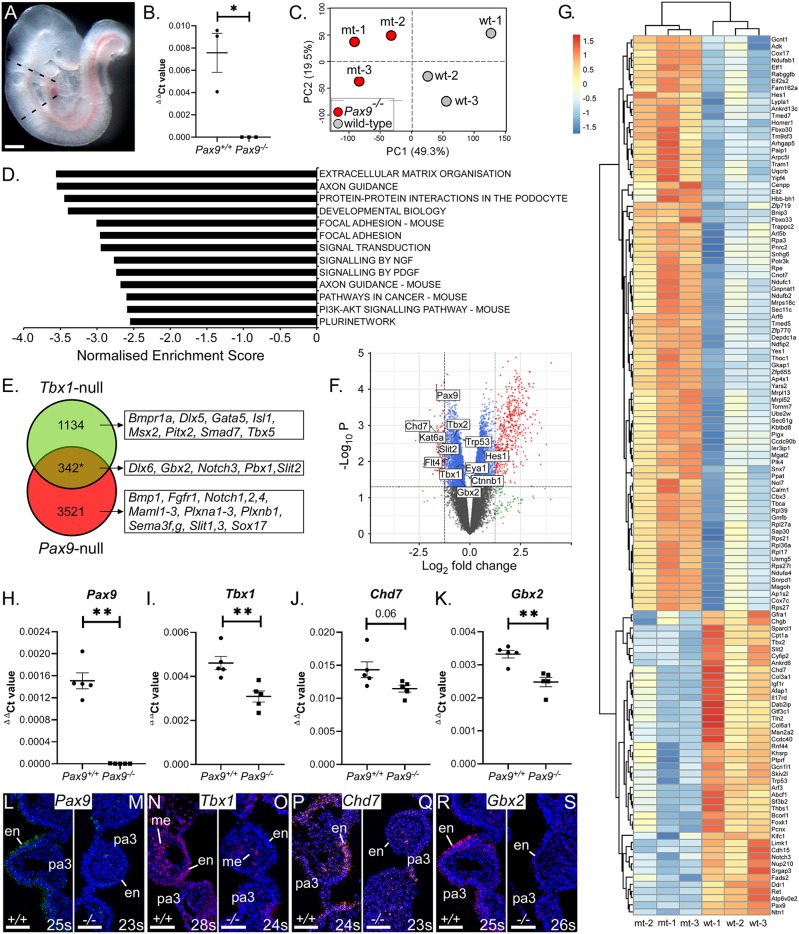
***Pax9* shares a genetic network with *Tbx1*.** (A) The pharyngeal arch region of control and *Pax9^–/–^* embryos at E9.5 (*n*=3 per genotype; 26-27 s) was dissected (indicated by dashed lines). Scale bar: 500 µm. (B) Total RNA was extracted, converted to cDNA and checked by qPCR. (C) Principle component analysis of RNA-seq data showed a clear separation between control and mutant samples (shown on PC2) and only slight biological variation across replicates (shown on PC1). (D) Gene set enrichment analysis identified signalling pathways predicted to be significantly downregulated in *Pax9^–/–^* embryos. All pathways shown have a familywise-error rate of *P*<0.001. (E) *Tbx1*-related genes (*n*=1476) were compared with those genes significantly differentially expressed (DE) in *Pax9^–/–^* embryo tissue (*n*=3863, adjusted *P*≤0.1) giving 342 genes common to both datasets (*P=0.0029, as assessed by a hypergeometric distribution test). Examples of genes in each cohort are shown. (F) Volcano plot highlighting *Tbx1*-interacting genes significantly DE in *Pax9^–/–^* embryos (based on adjusted *P*≤0.1). (G) Heat map showing colour-coded (red, up; blue, down) expression levels of DE genes between *Pax9^+/+^* (wt) and *Pax9^–/–^* (mt) embryos that are common to *Tbx1* DE genes (adjusted *P*≤0.05 are shown). (H-K) qPCR analysis confirmed that *Pax9* (H), *Tbx1* (I), *Chd7* (J) and *Gbx2* (K) were significantly reduced in *Pax9^–/–^* pharyngeal tissue at E9.5 (*n*=5 embryos per genotype). **P<0.01; two-tailed unpaired *t*-test (*Tbx1* and *Chd7*) and two-tailed Mann–Whitney (*Pax9* and *Gbx2*). (L-S) RNAscope *in situ* hybridisation showed a reduced expression of *Pax9* (M), *Tbx1* (O), *Chd7* (Q) and *Gbx2* (S) in the pharyngeal endoderm in *Pax9^–/–^* embryos at E9.5 (*n*=3 per genotype, 23-28 s). en, endoderm; me, mesoderm; pa, pharyngeal arch. Somite counts (s) are indicated. Scale bars: 50 µm.

To further investigate the role of *Pax9* in cardiovascular development, we looked at the effect of *Pax9* loss on the pharyngeal arch transcriptome at E9.5 (Fig. 5A-C). Analysis of the RNA-seq data identified 3863 significant differentially expressed genes (Table S1). Gene set enrichment analysis revealed several significantly downregulated pathways, including ‘extracellular matrix organisation’ and ‘axon guidance’ (Fig. 5D). Interestingly, *Tbx1* was significantly reduced in *Pax9^–/–^* embryos at E9.5, and this was confirmed by qPCR (Fig. 5I). We also confirmed that *Pax9* expression was reduced in *Tbx1*-null embryos (Fig. S2). As *Tbx1* expression is crucial for 4th PAA morphogenesis (Lindsay et al., 2001; Zhang et al., 2005), these data suggested that *Pax9* may function together with *Tbx1* rather than being downstream of it. We therefore compiled a list of all the genes reported to be significantly differentially expressed in *Tbx1*-null embryos from published microarray (Ivins et al., 2005; Liao et al., 2008; van Bueren et al., 2010) and mouse genetic interaction studies, giving 1476 unique *Tbx1*-related genes. These were then compared with all genes significantly changed in *Pax9^–/–^* embryo pharyngeal arch tissue, giving 342 genes that were common to both datasets (Fig. 5E-G; Fig. S3; Table S1) and suggesting that *Tbx1* and *Pax9* may share a genetic network. Among these common genes were *Chd7* and *Gbx2*, which have been shown to interact with *Tbx1* in mice (Calmont et al., 2009; Randall et al., 2009), and were also reduced in *Pax9*-null tissue by qPCR and *in situ* hybridisation (Fig. 5H-S).

**Fig. 6.**
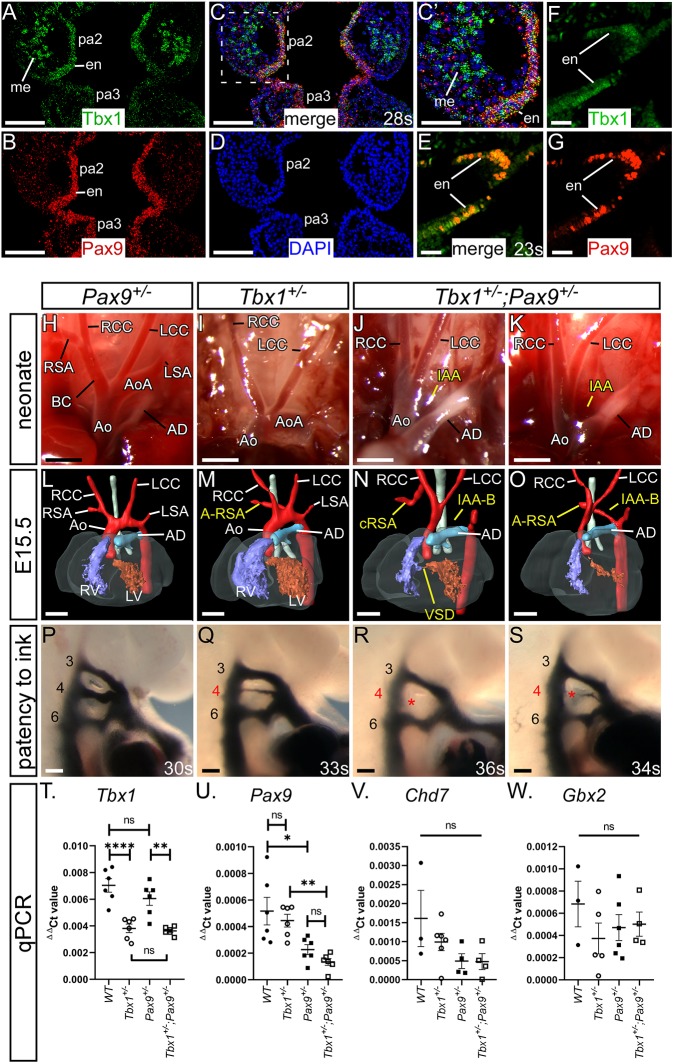
***Tbx1*;*Pax9* double heterozygous embryos have 4th PAA defects.** *Tbx1* and *Pax9* expression in the pharyngeal arches was examined at E9.5. (A-D) RNAScope *in situ* hybridisation expression of *Tbx1* in the 2nd and 3rd pharyngeal arch (pa), endoderm (en) and mesoderm (me; A), and *Pax9* expression in the endoderm only (B). The signals overlap in the endoderm (C, and higher power in C′). (D) DAPI staining. (E-G) Immunostaining for Tbx1 and Pax9 (E) demonstrates protein colocalisation within cells of the pharyngeal endoderm. Single staining for Tbx1 (F) and Pax9 (G) is shown. (H-K) Neonates from a *Tbx1^+/–^* and *Pax9^+/–^* intercross were recovered and the aortic arches examined. (H) Arch arteries were normal in all *Pax9^+/–^* neonates (*n*=10). (I) *Tbx1^+/–^* neonates (*n*=6) often presented with A-RSA, inferred by the absence of the brachiocephalic and right subclavian arteries. (J,K) All *Tbx1^+/–^; Pax9^+/–^* neonates (*n*=9) died within 24 h of birth with IAA and/or A-RSA. (L-O) 3D reconstructions of E15.5 embryo hearts from MRI datasets. (L) *Pax9^+/–^* hearts were normal (*n*=15). (M) *Tbx1^+/–^* hearts (*n*=19) frequently displayed 4th PAA defects such as A-RSA. (N,O) All *Tbx1^+/–^; Pax9^+/–^* embryos (*n*=20) presented with a 4th PAA-derived defect such as IAA-B, A-RSA and/or cervical right subclavian artery (cRSA). (P-S) Intracardiac ink injections into E10.5 embryos (27-38 s). (P) PAAs in control embryos were normal (*n*=10). (Q) In *Tbx1^+/–^* embryos (*n*=8) the 4th PAAs were often hypoplastic. (R,S) In *Tbx1^+/–^;Pax9^+/–^* embryos (*n*=9), the 4th PAAs were frequently bilaterally absent. Ao, aorta; AoA, aortic arch; AD, arterial duct; BC, brachiocephalic; LCC, left common carotid artery; LSA, left subclavian artery; LV, left ventricle; RCC, right common carotid artery; RSA, right subclavian artery; RV, right ventricle. Somite counts (s) are indicated. Scale bars: 2 mm in H-K; 500 μm in L-O; 100 μm in A-D,P-S; 50 µm in E-G. (T-W) qPCR analysis in wild-type, *Tbx1^+/–^*, *Pax9^+/–^* and *Tbx1^+/–^;Pax9^+/–^* embryos (*n*≥3 per genotype, somite range 23-27). (T) *Tbx1* levels were significantly reduced in *Tbx1^+/–^* and *Tbx1^+/–^;Pax9^+/–^* embryos. (U) *Pax9* levels were significantly reduced in *Pax9^+/–^* and *Tbx1^+/–^;Pax9^+/–^* embryos. There was no significant difference in *Tbx1* (T), *Pax9* (U), *Chd7* (V) or *Gbx2* (W) levels between single heterozygotes and *Tbx1^+/–^;Pax9^+/–^* embryos. One-way ANOVA with Tukey's multiple comparison test. ns, not significant. *P<0.05, **P<0.01, ****P<0.0001.

To investigate a potential genetic interaction *in vivo* between *Tbx1* and *Pax9*, we confirmed that both genes were co-expressed in the pharyngeal endoderm (Fig. 6A-G). We then crossed *Tbx1^+/–^* mice (Jerome and Papaioannou, 2001) with *Pax9^+/–^* mice to create *Tbx1^+/–^;Pax9^+/–^* double heterozygotes. Genotype analysis of 168 pups at weaning revealed that only two *Tbx1^+/–^;Pax9^+/–^* mice were alive, a highly significant deviation from the 42 expected of each genotype from this cross (*P*=1.4×10^−12^; Table S2). To identify the timing of lethality, neonates were observed from the day of birth with all *Tbx1^+/–^*;*Pax9^+/–^* mutants dying during the first 24 h. Examination of neonatal aortic arch arteries revealed that all controls were normal (Fig. 6H), while *Tbx1^+/–^* neonates frequently presented with A-RSA (Fig. 6I; Table 1). All dead *Tbx1^+/–^*;*Pax9^+/–^* neonates had 4th PAA-derived defects, including IAA (Fig. 6J,K; Table 1).

Having established postnatal cardiovascular defects, we next analysed E15.5 *Tbx1^+/–^*;*Pax9^+/–^* embryos by MRI (Fig. 6L-O). All wild-type and *Pax9^+/–^* embryos were normal. Of the *Tbx1^+/–^* embryos examined, a high proportion had some form of 4th PAA-derived defect, including IAA and A-RSA (Fig. 6M; Table 1). All *Tbx1^+/–^*;*Pax9^+/–^* embryos examined, however, had some form of 4th PAA-derived defect (Fig. 6N,O; Table 1) with the incidence of IAA being significantly increased compared with *Tbx1^+/–^* embryos (*P*<0.001). The incidence of perimembranous VSD was also significantly increased (*P=*0.04), but none of the embryos examined displayed outflow tract defects or bicuspid aortic valve, although an abnormal thymus was frequently observed (Fig. S4; Table S3). *Tbx1^+/–^*;*Pax9^+/–^* embryos at E10.5 were injected intracardially with ink (Fig. 6P-S) and the patency of the developing 4th PAAs was then compared between *Tbx1^+/–^* and *Tbx1^+/–^*;*Pax9^+/–^* embryos (Table 2). All *Tbx1^+/–^* embryos displayed a predominantly hypoplastic 4th PAA defect, with 50% of embryos having a unilateral, and 50% a bilateral, defect. In *Tbx1^+/–^*;*Pax9^+/–^* embryos, a significant increase in bilateral aplasia of the 4th PAAs (*P=*0.004) was found, but no defects of PAAs 1, 2, 3 or 6 were seen. RNA analysis showed that *Tbx1*, *Pax9*, *Chd7* and *Gbx2* RNA levels were not significantly changed between the respective single heterozygotes and *Tbx1^+/–^*;*Pax9^+/–^* pharyngeal arches (Fig. 6T-W).

### *Tbx1* and *Pax9* interact cell autonomously in the pharyngeal endoderm for 4th PAA formation

**Fig. 7.**
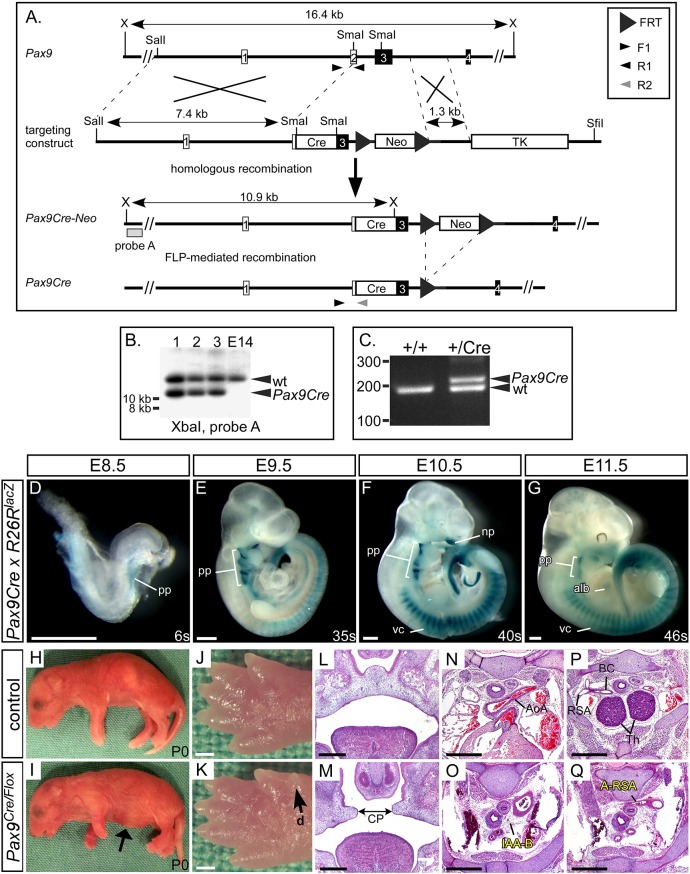
**Generation of the *Pax9Cre* mouse.** (A) A *Pax9Cre* targeting vector was created with a *Cre* cassette inserted into the SmaI restriction sites found in exons 2 and 3 of the *Pax9* gene. For transformation, the vector was linearised with SfiI. (B) Correctly targeted ES cells were identified by Southern blotting on XbaI-digested genomic DNA. (C) The derived *Pax9Cre-Neo* mice were crossed with *FLPe*-expressing mice to remove the neomycin cassette and create *Pax9Cre* mice, which were genotyped using the indicated primers. (D-G) *Pax9Cre* mice were mated with *R26R^lacZ^* reporter mice and embryos were stained with X-Gal at E8.5 (*n*=3), E9.5 (*n*=7), E10.5 (*n*=6) and E11.5 (*n*=5). Positive staining in regions known to express Pax9 was observed (alb, anterior limb bud; np, nasal process; pp, pharyngeal pouch; vc, vertebral column). (H-Q) *Pax9Cre* and *Pax9^flox/flox^* mice were crossed to create *Pax9^+/flox^* (control) and *Pax9^Cre/flox^* (mutant) mice. All *Pax9^Cre/flox^* neonates died on the day of birth and presented with the typical *Pax9*-null phenotype (*n*=5), including a bloated abdomen (I, arrow) and pre-axial digit duplication (d) in the hind limb (K, arrow). Sections of E14.5-E15.5 embryos (*n*=6) showed cleft palate (CP; M), absent thymus and cardiovascular defects (O,Q). AoA, aortic arch; BC, brachiocephalic; Th, thymus. Somite counts (s) are indicated. Scale bars: 500 µm.

**Fig. 8.**
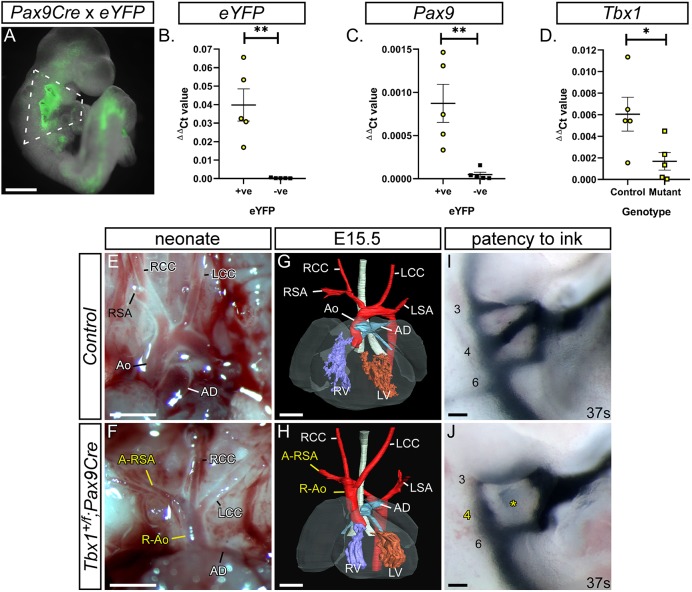
**Conditional deletion of *Tbx1* from the pharyngeal endoderm.** (A) *Pax9Cre* drives expression of the eYFP reporter gene in the pharyngeal endoderm at E9.5. Scale bar: 500 µm. (B-D) Flow sorting and qPCR of eYFP-positive and -negative pharyngeal arch cells from *Pax9Cre;eYFP* control and *Tbx1^+/flox^;Pax9Cre* E9.5 embryos (*n*=5 of each; dissected as shown in A). From control embryos, the eYFP-positive cells (yellow circles) were significantly enriched for *eYFP* (B) and *Pax9* (C) compared with eYFP-negative cells (black squares). (D) There was a significant reduction in *Tbx1* levels in *Tbx1^+/flox^;Pax9Cre* (mutant) eYFP-positive cells (yellow squares) compared with controls (yellow circles). *P<0.05, **P<0.01 (two-tailed unpaired *t*-test). (E-J) Cardiovascular defects in *Tbx1^+/flox^;Pax9Cre* mice. (E,F) Neonates were collected shortly after birth and the aortic arch arteries examined. (E) Control neonates (*n*=39 wild-type, *n*=43 *Pax9Cre* and *n*=33 *Tbx1^+/flox^*) all had normal arch arteries. (F) *Tbx1^+/flox^;Pax9Cre* mutants (*n*=8) had cardiovascular defects such as a right-sided aorta (R-Ao) and A-RSA. Scale bars: 1 mm. (G,H) Embryos at E13.5-E15.5 were analysed by imaging (*n*=26 mutants). (G) Control embryo with normal cardiovascular system. (H) In the mutant there is a vascular ring formed by a R-Ao and an A-RSA. Scale bars: 500 µm. (I,J) Embryos collected at E10.5 (36-43 s) were injected intra-cardially with ink. (I) In *Tbx1^+/flox^* control embryos (*n*=15), PAAs 3-6 were patent to ink. (J) In *Tbx1^+/flox^;Pax9Cre* mutant embryos (*n*=17), the 4th PAA was frequently absent (asterisk). Somite counts (s) are indicated. Scale bars: 100 µm. Ao, aorta; AD, arterial duct; LCC, left common carotid artery; LSA, left subclavian artery; LV, left ventricle; RCC, right common carotid artery; RSA, right subclavian artery; RV, right ventricle. The somite numbers given in the legend reflect the range analysed for the whole study. The figure contains representative images only.

The presumed site of the *Tbx1-Pax9* interaction is the pharyngeal endoderm, and to demonstrate a cell-autonomous interaction in this tissue, we crossed *Tbx1^flox^* mice (Xu et al., 2004) with a novel *Pax9Cre* transgenic mouse (Fig. 7A-C). This *Pax9Cre* strain has the *Cre* recombinase gene inserted into the *Pax9*-coding region and effectively results in a *Pax9*-null allele. The *Pax9Cre* allele functions as expected by activating *R26R^lacZ^* expression specifically in the pharyngeal endoderm from E9.5 to E11.5 (Fig. 7D-G; Fig. S5), and *Pax9Cre;Pax9^flox^* neonates (i.e. *Pax9*-null) die at birth with the typical *Pax9^–/–^* phenotype (Fig. 7H-Q). To test the specificity of the *Pax9Cre* allele, the pharyngeal arches of *Pax9Cre;eYFP* E9.5 embryos (Fig. 8A) were dissociated into single cells and flow-sorted into eYFP-positive and -negative populations. The eYFP-positive population was significantly enriched for *eYFP* and *Pax9* transcripts (Fig. 8B,C). *Tbx1^flox^* and *Pax9Cre* mice were intercrossed to create *Tbx1^+/ flox^;Pax9Cre* embryos, which are double heterozygous for each gene but only in the *Pax9* expression domain. A significant reduction in *Tbx1* RNA levels was observed in *eYFP*-positive flow-sorted pharyngeal endoderm cells from conditionally deleted E9.5 mutant embryos compared with *Pax9Cre;eYFP* controls (Fig. 8D). Litters from a *Tbx1^+/flox^* and *Pax9Cre* cross were collected on the day of birth; a significant reduction in the expected number of *Tbx1^+/flox^;Pax9Cre* mutants was found (*P=*2.4×10^−5^; Table S4) and 62.5% of these mutants displayed an arch artery defect (Fig. 8F; Table 1). We then collected embryos (E13.5-E15.5) and analysed them by MRI or µCT, which revealed that 38.5% of mutants presented with an arch artery defect (Fig. 8H; Table 1) and an abnormal thymus (Fig. S4; Table S3). We also collected embryos at E10.5 and injected them intracardially with ink to visualise the PAAs. All *Tbx1^+/flox^* control embryos analysed had normal PAAs, with all three vessels being patent to ink and of a similar diameter, while the majority of *Tbx1^+/flox^;Pax9Cre* mutant embryos presented with some form of 4th PAA defect (Fig. 8J; Table 2).

In summary, our data show that *Pax9* is crucial for cardiovascular development, and when lost the PAAs and outflow tract fail to develop correctly, resulting in postnatal death. The 4th PAAs do not form and the 3rd PAAs collapse with reduced recruitment from vascular SMC. Our data also demonstrate that *Tbx1* and associated genes are deregulated in *Pax9*-null embryos, and *Tbx1* and *Pax9* function together in the pharyngeal endoderm for 4th PAA morphogenesis.

## DISCUSSION

### Loss of *Pax9* results in complex cardiovascular defects

*Pax9* plays a key role in cardiovascular development, as *Pax9^–/–^* neonates and embryos display a range of cardiovascular defects. Gene mutations and chromosomal deletions that include *PAX9* have been identified in individuals with cardiovascular abnormalities and other conditions (Table S5), suggesting that *PAX9* has potential importance in human cardiovascular development. Of these, one individual with a 105 kb hemizygous deletion of 14q13 presented with IAA, a hypoplastic aorta, bicuspid aortic valve and a ventricular septal defect (Santen et al., 2012), all of which appear with high penetrance in mouse *Pax9*-null embryos. This small deletion removed only *PAX9*, *NKX2-1* and *NKX2-8*, although deletion of either *Nkx2* gene in mice does not result in any cardiovascular defects (Kimura et al., 1996; Pabst et al., 2003). It is therefore possible that hemizygous deletion of *PAX9* in humans can recapitulate, in part, the mouse knockout phenotype.

### *Tbx1* and *Pax9* interact in 4th PAA development

Published microarray studies investigating the transcriptome of *Tbx1*-null embryos have implicated *Pax9* as a downstream target of *Tbx1* (Ivins et al., 2005; Liao et al., 2008). Further evidence of crosstalk between *Tbx1* and *Pax9* has been suggested via the interaction of *Ripply3* (Okubo et al., 2011). This protein, which is a Tbx1 repressor, has been shown to prevent Tbx1 activation of luciferase from a *Pax9* promoter construct, and *Pax9* expression in the pharyngeal endoderm is increased in *Ripply3*-null embryos suggesting that *Tbx1* may be upstream of *Pax9*. *Ripply3* levels were not significantly changed in our *Pax9*-null pharyngeal arch transcriptome data, but *Tbx1* levels were significantly reduced, indicating that perhaps *Pax9* and *Tbx1* may function together rather than work in a hierarchal pathway. This is further supported by analysis of *Tbx1* and *Pax9* RNA levels in *Tbx1^+/–^;Pax9^+/–^* embryos, which were not significantly changed compared with levels seen in the single heterozygotes. Further analysis identified that many genes differentially expressed in *Tbx1*-null embryos, or shown to interact with *Tbx1* in mouse models, were also significantly differentially expressed in *Pax9*-null embryos, e.g. *Chd7* and *Gbx2*. Mice heterozygous for *Chd7* have abnormal 4th PAA derivatives and a combined heterozygosity with *Tbx1* results in an increased incidence of 4th PAA defects (Randall et al., 2009). *Gbx2*-null embryos display cardiovascular defects (Byrd and Meyers, 2005), and *Tbx1* and *Gbx2* have also been shown to genetically interact in PAA development (Calmont et al., 2009). Although both these *Tbx1* targets have been demonstrated to interact in the pharyngeal ectoderm, they are also expressed in the pharyngeal endoderm and could therefore interact with *Pax9* either directly or indirectly in this tissue. As our data strongly suggested that *Pax9* may interact with *Tbx1* and its related genes in the morphogenesis of the PAAs, we therefore looked for a genetic interaction between *Tbx1* and *Pax9* in mice. We found that all mice examined (embryos and neonates) double heterozygous for each gene (i.e. *Tbx1^+/–^*;*Pax9^+/–^*) displayed some defect associated with abnormal development of the 4th PAA, most notably IAA-B. Although hypoplasia or absence of the 4th PAAs is a feature in mouse embryos heterozygous for *Tbx1* (Lindsay et al., 2001), this phenotype was significantly more prevalent in *Tbx1^+/–^*;*Pax9^+/–^* mice, and much higher than observed in *Tbx1^+/–^* embryos. It therefore appears that combining one *Pax9*-null allele with one *Tbx1*-null allele exacerbates the *Tbx1^+/–^* phenotype.

We used our novel *Pax9Cre* mouse to conditionally delete *Tbx1* from the pharyngeal endoderm, while simultaneously creating double haploinsufficiency for the two genes in this tissue. A highly significant reduction in the number of *Tbx1^+/flox^;Pax9Cre* neonates born was observed, indicating that heterozygous loss of *Tbx1* and *Pax9* from the pharyngeal endoderm has a major impact on postnatal survival, with almost two-thirds of the recovered neonates examined having identifiable cardiovascular defects. A similar proportion of affected mutants was found at foetal stages. A vascular ring structure was observed in *Tbx1^+/flox^;Pax9Cre* mutants (Fig. 8H), similar to that described in *Lgdel/+* mice, which are chromosomally engineered to be hemizygous for *Tbx1* (Merscher et al., 2001), and also in *Six1;Eya1* mutant embryos (Guo et al., 2011a). *Eya1* was significantly reduced in *Pax9*-null pharyngeal arch tissue. When embryos were examined at mid-embryogenesis, however, we found that almost all displayed 4th PAA defects. When compared with our *Tbx1^+/–^*;*Pax9^+/–^* embryo data it was apparent that there was no significant difference in the incidence of bilateral 4th PAA defects between *Tbx1^+/flox^;Pax9Cre* and *Tbx1^+/–^*;*Pax9^+/–^* embryos. This indicates that the conditional heterozygous deletion of *Tbx1* from the pharyngeal endoderm, in the context of *Pax9* heterozygosity, has the same effect on 4th PAA morphogenesis as seen in the global *Tbx1^+/–^*;*Pax9^+/–^* embryos at E10.5. Although the ink injection data from *Tbx1^+/flox^;Pax9Cre* embryos matches that seen in *Tbx1^+/–^*;*Pax9^+/–^* embryos, and shows that heterozygous loss of *Tbx1* from the pharyngeal endoderm severely affects PAA morphogenesis, there is a discrepancy in the cardiovascular phenotype observed at foetal and neonatal stages that does not reflect that seen at mid-embryogenesis, nor that observed in *Tbx1^+/–^*;*Pax9^+/–^* embryos. It is well documented, however, that the *Tbx1^+/–^* 4th PAA phenotype does recover during development (see Table S6). It therefore appears that a conditional deletion of *Tbx1* from the pharyngeal endoderm in the context of *Pax9* haploinsufficiency replicates the *Tbx1* heterozygous arch artery phenotype in terms of a highly penetrant 4th PAA defect at mid-embryogenesis that partially recovers by the foetal stage. Our data, however, do raise the question of whether we are seeing the effect of a pharyngeal endoderm-specific loss of *Tbx1* with *Pax9Cre* or a genetic interaction occurring between *Tbx1* and *Pax9* in the pharyngeal endoderm? Conditional deletion experiments have been performed in transgenic mice to identify the role of *Tbx1* in specific tissues and have examined the effect of *Tbx1* heterozygosity in the pharyngeal mesoderm and/or epithelium for 4th PAA development (see Table S7). When compared, our data clearly show that the 4th PAA phenotype is much more penetrant than that seen in embryos only heterozygous for *Tbx1* in the pharyngeal epithelia. We therefore conclude that we are showing a strong genetic and cell-autonomous interaction between *Tbx1* and *Pax9* within the pharyngeal endoderm that impacts on 4th PAA morphogenesis. Moreover, and in support of a phenotype extending beyond what is expected from a *Tbx1* heterozygous phenotype, our *Tbx1^flox^;Pax9Cre* mice display a high incidence of perinatal lethality, a feature not previously described in *Tbx1^+/–^* mice. Interestingly, the homozygous deletion of *Tbx1* from the pharyngeal endoderm using *Sox17Cre* mice resulted in pharyngeal arch aplasia but not in a common arterial trunk, although IAA was observed (Jackson et al., 2014). This phenotype is similar to that seen in our *Tbx1;Pax9* mutants but is distinct from either *Tbx1* and *Pax9* constitutive null mutants.

### Pax9 signalling from the pharyngeal endoderm is required for 3rd PAA maintenance

The mammalian PAAs develop symmetrically in a cranial to caudal sequence, and then subsequently remodel to form the typical asymmetric aortic arch artery system seen in the adult (Bamforth et al., 2013; Hiruma et al., 2002). In normal PAA development, the 1st artery forms first, followed by the 2nd, but these two vessels rapidly remodel and contribute to capillary beds and the mandibular and hyoid arteries. In some *Pax9^–/–^* embryos, these vessels aberrantly persisted into E10.5 and E11.5, and may contribute to the anomalous arrangement of the aortic arch arteries when the 3rd PAAs collapse. In normal development, the 3rd PAAs will form part of the common and proximal internal carotid arteries, which elongate as the embryo grows. The carotid duct involutes and the dorsal aorta anterior to this segment will form the distal internal carotid artery, with the external carotid artery derived from the proximal parts of the 1st and 2nd PAA (Hiruma et al., 2002). The persistence of the 1st and/or 2nd PAAs in *Pax9^–/–^* embryos, coupled with the failure of the 3rd PAAs to be maintained and the carotid duct to involute, results in the external carotid arteries rising directly from the aorta, and the internal carotid arteries from the anterior dorsal aortae. This phenomenon has been described clinically (Roberts and Gerald, 1978). Although reduced numbers of NCCs were found in the caudal pharyngeal arches at E10.5, migration, apoptosis or proliferation of NCCs did not appear to be impaired in *Pax9*-null embryos. It is well recognised that NCCs differentiate into the SMCs that are important for the stabilisation of the remodelled PAAs (Hutson and Kirby, 2003) and it is likely that the failure of SMCs to envelop the 3rd PAAs in *Pax9*-null embryos results in the collapse of this vessel. This has also been described in *Hoxa3*-null embryos (Kameda, 2009) and reduced NCC migration in genetic mouse and surgical chick models also leads to defects of the 3rd PAAs (Bradshaw et al., 2009; Epstein et al., 2000; Franz, 1989; Nishibatake et al., 1987). We were unable to look for a similar occurrence in the 4th PAAs, as this fails to form in the majority of *Pax9*-null embryos. Signalling between the pharyngeal endoderm and NCC does occur (Graham et al., 2005) so it is therefore possible that signals emanating from the pharyngeal endoderm under Pax9 control are necessary to influence the differentiation of NCCs into SMCs; without these signals, the 3rd PAAs are not supported during the remodelling phase of arch artery development and they collapse. The top two GSEA pathways identified from the RNA-seq data were ‘extracellular matrix organisation’ and ‘axon guidance’, and these could be relevant for a NCC-derived phenotype, particularly as the extracellular matrix is known to be crucial for the migration of NCCs (Henderson and Copp, 1997; Perris and Perissinotto, 2000) and the slit, ephrin, semaphorin and plexin genes listed under ‘axon guidance’ are usually associated with NCC expression or migration in cardiovascular development (Epstein et al., 2015; Kirby and Hutson, 2010; Sun et al., 2018). There is, however, an alternative explanation for the collapse of the 3rd PAAs. Haemodynamic force plays a major role in artery development (Culver and Dickinson, 2010; Yashiro et al., 2007), so it is possible that the 3rd PAA defect is a consequence of increased blood flow to the persistent 1st/2nd PAAs, which prevents sufficient blood flow to the 3rd PAAs, resulting in impairment of flow-induced arteriogenesis. This may also be an explanation for the failure of the 4th PAAs to form. A reduced haemodynamic force would also explain the reduction in SMC recruitment as this is also dependent on blood flow (Padget et al., 2019). Although endothelial cells were present within the 4th pharyngeal arch of *Pax9*-null embryos, they apparently failed to condense into tubes, a feature that has also been described in both *Tbx1*-null and *Gbx2-*null embryos (Byrd and Meyers, 2005; Calmont et al., 2009).

The double outlet right ventricle phenotype observed at E15.5 in *Pax9*-null embryos probably arises from the marked outflow tract rotation defect due to a change in the aortic to pulmonary valve axis angle (Bostrom and Hutchins, 1988; Lomonico et al., 1986), which is also observed in *Pax3* and *Pitx2c* mutant hearts (Bajolle et al., 2006).

**Fig. 9.**
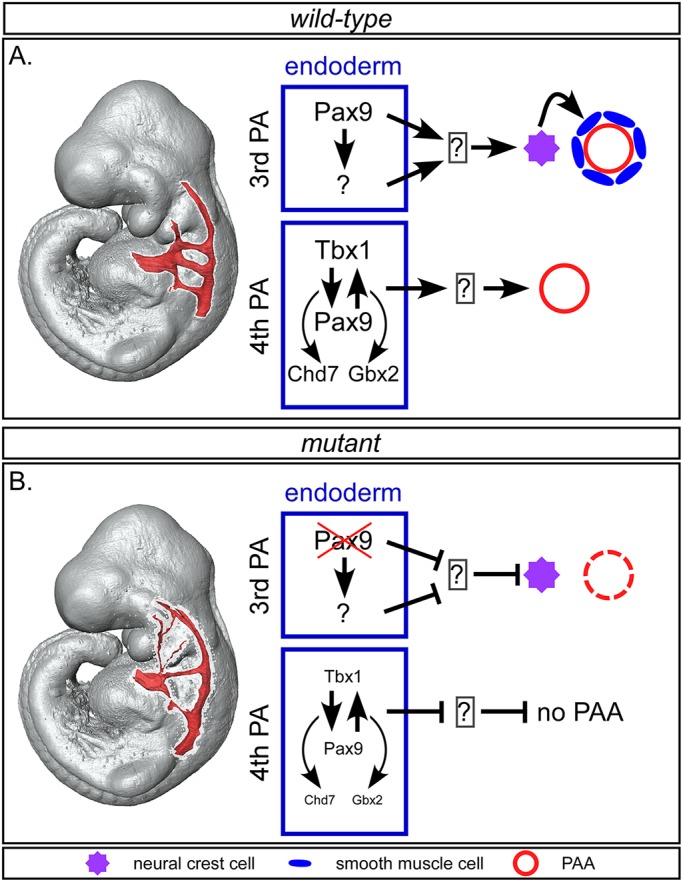
**Proposed model for role of Pax9-mediated signalling during pharyngeal arch artery development.** (A) In wild-type embryos, *Pax9* expression in the 3rd pharyngeal arch endoderm induces neural crest cells (NCCs), either directly or indirectly, to differentiate into the SMCs that form around the endothelium of the remodelling 3rd PAAs. In the 4th arch endoderm, *Tbx1* and *Pax9* function together to, most likely indirectly, control formation of the 4th PAAs, potentially through collaboration with *Chd7* and *Gbx2*. (B) In *Pax9*-deficient embryos, signals from the 3rd pharyngeal arch endoderm are compromised and NCC differentiation into SMCs is impaired, resulting in the collapse of the 3rd PAAs by E11.5. In *Tbx1-Pax9* double heterozygous embryos, reduced levels of *Tbx1* and *Pax9* in the 4th pharyngeal arch endoderm are insufficient to allow the 4th PAAs to form.

In summary, Pax9 expression in the pharyngeal endoderm has regional (anterior/posterior) roles in regulating the morphogenesis of the PAAs (Fig. 9). In the 3rd pharyngeal arch, Pax9 is important for controlling NCC differentiation into the SMCs that support the remodelling vessel, whereas in the 4th pharyngeal arch endoderm, the interaction of *Pax9* with *Tbx1* and its targets is crucial for the formation of the 4th PAAs.

## MATERIALS AND METHODS

### Mice

The mice used in this study have previously been described: *Pax9^+/–^* (Peters et al., 1998), *Pax9^flox^* (Kist et al., 2007), *Tbx^+/–^* (Jerome and Papaioannou, 2001), *Tbx1^flox^* (Xu et al., 2004), *Wnt1Cre* (Danielian et al., 1998), *R26R^eYFP^* (Srinivas et al., 2001), *R26R^lacZ^* (Soriano, 1999) and *FLPe* (Dymecki, 1996). All mice were maintained on a C57Bl/6J genetic background. All studies involving animals were performed in accordance with UK Home Office Animals (Scientific Procedures) Act 1986.

### Generation of *Pax9Cre* mice

The *Pax9Cre* targeting construct was designed to replace a 1.06 kb section of the *Pax9* gene, spanning the second half of exon two, containing the start codon, and the first half of the 3rd exon, containing the paired box and octapeptide motif, with a promoterless *Cre* recombinase gene preceded by a consensus Kozak sequence, in the pPNT4 targeting vector (Conrad et al., 2003), using standard molecular biology methods (Fig. 7A). The 1.3 kb right homology arm, generated as previously described (Kist et al., 2005), was inserted between the FRT-flanked PGK-Neomycin cassette and the HSV-Thymidine Kinase cassette of pPNT4. The unwanted loxP site of pPNT4 was removed by XbaI partial digestion, followed by Klenow treatment and re-ligation. The left homology arm was generated as a 7.4 kb SalI to SmaI fragment of the *Pax9* genomic region that included exon 1 and the first half of the second exon, and was inserted 5′ of the *Cre* recombinase gene. The targeting vector was linearized with SfiI and mouse ES cells were targeted and selected as previously described (Kearns et al., 2013). Southern blot analysis was performed according to standard methods following XbaI digest of genomic DNA. An external southern probe was used to verify integration of the targeting cassette in the 5′ region (Fig. 7B) and PCR was used to validate integration of the 3′ region (not shown). The FRT-flanked PGK-neomycin cassette was removed by crossing the derived *Pax9Cre-Neo* mice with *FLPe* mice (Dymecki, 1996) to create *Pax9Cre* mice, which were then backcrossed to strain C57Bl/6J for over six generations. Genotyping by multiplex PCR [primers: F1-ACTCAAGCCTCTTTCAGCCC (common), R1-TTGTTCTCACTGAGCCGGCCTGT (Pax9 exon 2) and R2-GTTGCATCGACCGGTAATGC (Cre)] were used to simultaneously identify the wild-type and *Pax9Cre* alleles (Fig. 7C).

### Breeding

Male and female mice were mated and the detection of a vaginal plug the next morning considered to be E0.5. Pregnant females were culled on the required day and embryos collected. Embryos at E9.5-E11.5 were staged by somite counting.

### Imaging

Magnetic resonance imaging (MRI) was performed using a 9.4T MR system (Varian, US) as previously described (Bamforth et al., 2012; Schneider et al., 2004) on embryos at E15.5. High Resolution Episcopic Microscopy (HREM) and micro-computed tomography (µCT) techniques have been described in detail elsewhere (Degenhardt et al., 2010; Geyer et al., 2009). All MRI, HREM and µCT images were converted into a volume dataset and segmented using Amira software (Thermo Fisher Scientific) to create 2- and 3-dimensional (3D) images. Structures were manually outlined using the label field function of Amira and surface rendered to produce the 3D images. Intra-cardiac ink injections were performed as described previously (Calmont et al., 2009). Aorta measurements were taken from the region just proximal to the point at which the ascending aorta becomes the aortic arch. Haematoxylin and Eosin staining, X-gal staining and *Pax9* whole-mount *in situ* hybridisation were performed using standard techniques. mRNA expression on sections was examined by *in situ* hybridisation using RNAscope Multiplex Fluorescent v2 Assay (Advanced Cell Diagnostics) following the manufacturer's instructions. Probe details given in Table S8. For proliferation assays, BrdU (Sigma-Aldrich) dissolved in PBS was administered by intraperitoneal injection at a dose of 50 mg/kg to pregnant dams 1 h before embryo collection. Immunohistochemistry was performed using standard techniques (antibody details are in Table S8) on paraformaldehyde-fixed embryo sections and fluorescently imaged on an Axioimager (Zeiss). To assess an apoptotic index within the pharyngeal arches, control and *Pax9^–/–^* embryos at E9.5 (*n*=3 per genotype; 24-28 somites) and E10.5 (*n*=3 per genotype; 30-35 somites) were examined following immunostaining with the anti-caspase 3 antibody. To assess a proliferative index within the NCC in the pharyngeal arches, control and *Pax9^–/–^* embryos expressing *Wnt1Cre* activated eYFP at E9.5 (*n*=3 per genotype; 23-28 somites) and E10.5 (*n*≥4 for each genotype; 31-39 somites) were examined following immunostaining using anti-BrdU and anti-GFP antibodies. All positively stained and DAPI-positive cells within the pharyngeal arches were counted and the ratio of positively stained cells over the total number of cells calculated. The average number of NCCs within the caudal pharyngeal arches was calculated at E9.5 (*n*=3 per genotype, 23-28 somites) and E10.5 (*n*=4 per genotype, 32-34 somites). For all cell counting experiments, at least three sections per embryo were assessed.

### Transcriptome analysis

Wild-type (*Pax9^+/+^*) and *Pax9*-null (*Pax9^−/−^*) embryos at E9.5 (*n*=3 of each genotype, stage-matched for 26-27 somites) were collected in ice-cold DEPC-PBS and the pharyngeal region, from the junction between the first and second pharyngeal arch to the base of the heart, was dissected free in RNA*later* (Sigma-Aldrich). Genotypes were identified by PCR from the embryo tails and/or yolk sac material. Total RNA was extracted using the QIAGEN Plus Micro kit with genomic DNA Eliminator columns, and total RNA eluted in 30 μl RNase-free water. RNA concentration and purity were assessed by NanoDrop spectrophotometry and Agilent Bioanalyzer with all samples producing a RIN of 10. Genomic DNA contamination was excluded by PCR for intronic regions (sensitivity 5 pg/μl). To confirm that the correct genotypes were processed, cDNA was made from 100 ng total RNA and a qPCR for *Pax9* performed (Fig. 5B). The total RNA samples were shipped on dry-ice to EMBLM-GeneCore for RNA-seq first-strand specific library preparation using Illumina TruSeq, and 100 bp paired end reads were generated from an Illumina HiSeq 2000 Sequencer. Quality control by FastQC suggested a small amount of adaptor contamination in each sample, so raw reads were trimmed of adaptor sequence with Trimmomatic (Bolger et al., 2014). Reads shorter than 36 bp after trimming were discarded, and reads whose pair had been discarded were also removed. Trimmed reads were further filtered to keep only uniquely aligned and non-redundant reads to remove a vast majority of pseudogenes caused by multi-mapping. In addition, to avoid bias due to imbalanced library sizes among conditions, all samples were sub-sampled towards the smallest sample (mt-663_1). The resulting reads were aligned to the Mouse Genome (Ensembl release 81, GRCm38) using the splice aware read aligner STAR 2 (star-2.5.2a-0, 20/01/2017) (Dobin et al., 2013). Finally, aligned reads were assigned to Ensembl genes using featureCounts (Liao et al., 2014) with default parameters. These count tables were imported into R and analysed using the package LIMMA (Ritchie et al., 2015) and voom function from Bioconductor (Gentleman et al., 2004). A gene set enrichment analysis tool (Subramanian et al., 2005) was used to predict signalling pathways that were affected when gene expression in the wild-type and the *Pax9* mutant samples was compared. All resulting pathways were filtered for a familywise-error rate (*P*<0.001) to correct for multiple hypotheses testing.

### Flow cytometry

The pharyngeal arch region of E9.5 *Pax9Cre;eYFP* control (*n*=5; 23-27 somites) and mutant *Tbx1^+/flox^;Pax9Cre;eYFP* (*n*=5; 24-29 somites) embryos was dissected free as described above and dissociated to single cells with Accumax (eBioscience) by incubating at 37°C for 30 min. The reaction was stopped by the addition of 10% foetal calf serum (FCS), and cells were washed in PBS and resuspended in 10% FCS. Cells were stained with propidium iodide. Fluorescence-activated cell sorting was performed on a Becton Dickinson FACS Aria II using a 100 µm nozzle and a sheath pressure of 20 psi. Single cells were gated using FSC-A versus SSC-A followed by FSC-A versus FSC-H and FSC-A versus SSC-W to remove any doublets. Live single cells were gated using propidium iodide versus FSC-A, and this population was finally gated on eYFP-positive and -negative cells and sorted into collection tubes.

### Quantitative real-time RT-PCR (qPCR)

RNA, from either dissociated pharyngeal arch cells or flow-sorted cells, was extracted using Trizol reagent (Invitrogen) combined with a Purelink RNA Mini Kit (Ambion) and on-column DNase1 treatment. RNA was eluted with 30 µl RNase-free water. Total RNA was converted to cDNA using a High-Capacity cDNA Reverse transcription kit (Applied Biosystems) and random hexamers. qPCR was performed using SYBR Green JumpStart Taq ReadyMix (Sigma-Aldrich) using the primers listed in Table S9. All qPCR reactions were performed in triplicate on a QuantStudio 7 Real-Time PCR System (Thermo Fisher Scientific). Data were analysed using the comparative Ct method (Schmittgen and Livak, 2008).

### Statistical analysis

A chi-squared test was used to compare genotype frequencies of litters, and Pearson's chi-squared test for associations was used to compare defect frequencies between the different genotypes. For analysis of qPCR data, aorta measurements and cell counts, datasets were tested for variance using the Shapiro-Wilk test (Prism 8.01 software, GraphPad). Two-tailed unpaired *t*-tests were performed on normally distributed data, and a two-tailed Mann–Whitney *U* non-parametric test was used for not normally distributed data. One-way ANOVA with Tukey's multiple comparison test was used to analyse qPCR data from four embryo genotypes. A hypergeometric distribution test was used to calculate a *P*-value for observing an overlap between two gene lists in R. Groups were considered significantly different when *P*<0.05 or *P*≤0.1 for RNA-seq data. No statistical methods were used to predetermine sample size, which were chosen based on previous experience to obtain statistical significance and reproducibility. No data points were excluded and all data collected from each individual experiment were used for analysis.

## Supplementary Material

Supplementary information
