# Supplementary material for: Pax9 is required for cardiovascular development and interacts with Tbx1 in the pharyngeal endoderm to control 4th pharyngeal arch artery morphogenesis
Source: Development. 2019 Sep 23;146(18):dev177618. doi: 10.1242/dev.177618 (PMC6765178; doi:10.1242/dev.177618)
Supplement: Supplementary information [file develop-146-177618-s1.pdf]

Supplementary Figures

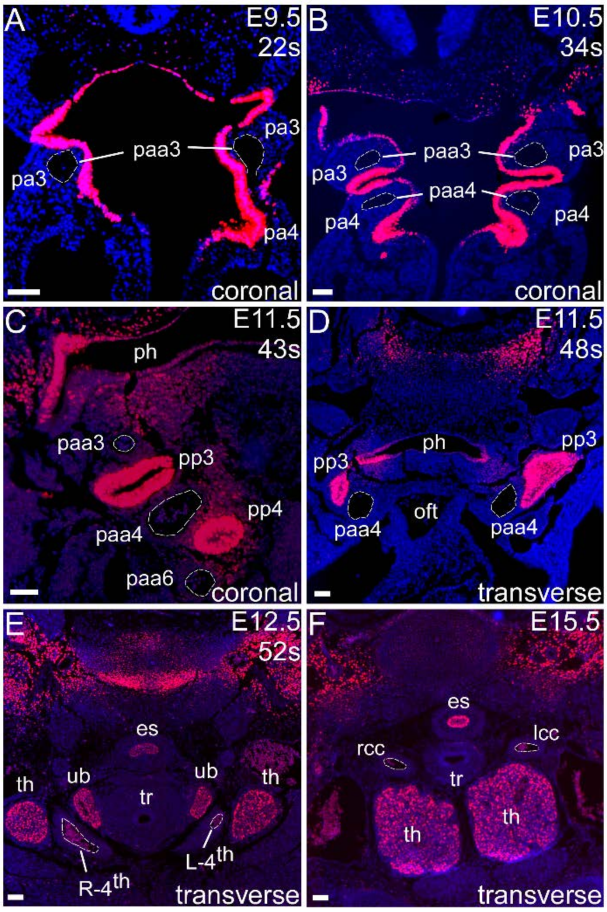

Figure S1. Pax9 expression during mouse pharyngeal development. A – C, coronal, and **D – F,** transverse sections of embryos at E9.5 (**A**), E10.5 (**B**), E11.5 (**C, D**), E12.5 (**E**) and E15.5 (**F**). Pharyngeal arch arteries (paa) are outlined. Pax9 is expressed in the pharyngeal endoderm at E9.5 and E10.5, and is subsequently expressed within the esophagus (es), developing thymus (th) and ultimobranchial bodies (ub) of the pharyngeal region. Somite counts (s) indicated. Abbreviations: L/R-4th, left/right 4th pharyngeal arch artery; lcc, left common carotid; oft, outflow tract; pa, pharyngeal arch; pp, pharyngeal pouch; ph, pharynx; rcc, right common carotid; tr, trachea. Scale, 100μm.

Development • Supplementary information

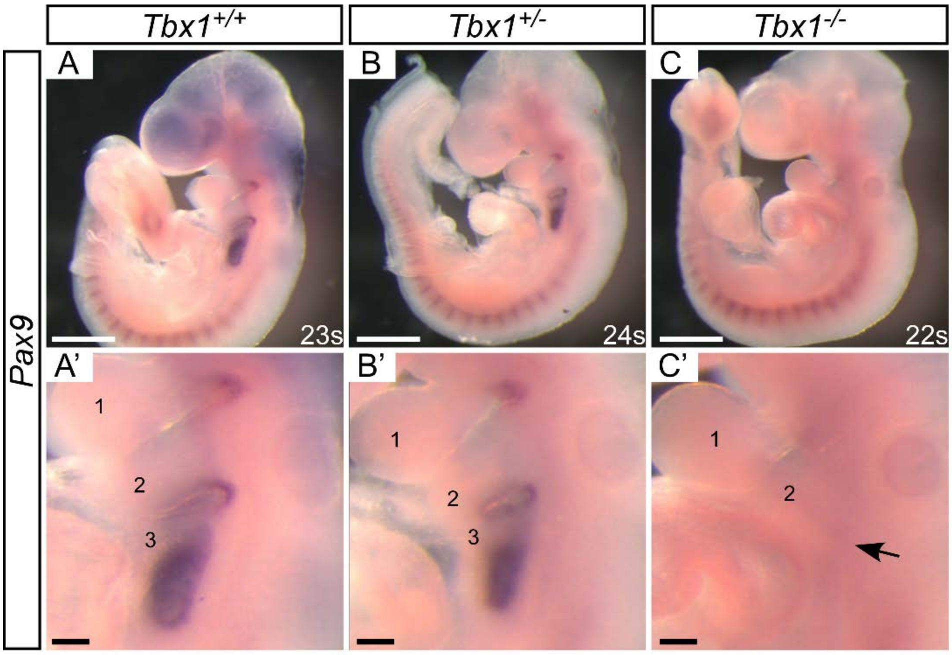

Figure S2. *Pax9* mRNA is reduced in the pharyngeal region of *Tbx1*$^{-/-}$ embryos. Whole-mount *in situ* hybridization using a *Pax9* riboprobe demonstrates that *Pax9* is specifically expressed in the pharyngeal endoderm and somites at E9.5 in wild-type (**A**) and *Tbx1*$^{+/-}$ (**B**) embryos. **C,** In *Tbx1*$^{-/-}$ embryos, however, *Pax9* levels are dramatically reduced from the pharyngeal endoderm (*arrow*). Somite counts (s) indicated. Scale: A – C, 500µm; A' – C', 100µm.

Development • Supplementary information

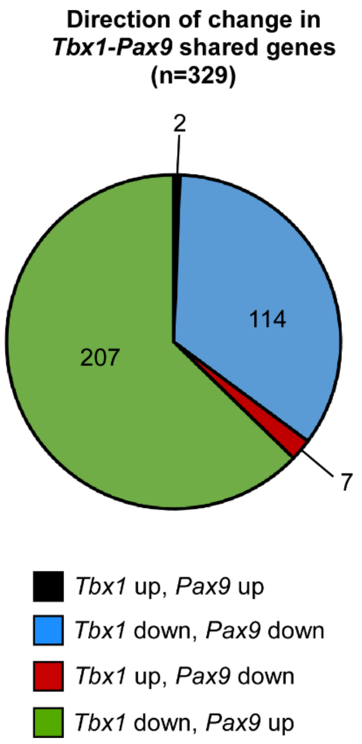

Gene up or down per dataset	Concordant		Discordant	
	***Tbx1* up, *Pax9* up**	***Tbx1* down, *Pax9* down**	***Tbx1* up, *Pax9* down**	***Tbx1* down, *Pax9* up**
n	2	114	7	207
Examples of genes in each category	*Cisd2, CxCl13*	*Aldh1a1, Gbx2, Gli3, Myh9, Notch3, Pax9, Pbx1, Tbx1*	*Col3a1, Col6a1, Kif1b, Sparcl1, Vtn*	*Bmp5, Bnip3, Cdkn1b, Cldn12, Dlx6, Fgfr1op2, Hes1*

Figure S3. Relationship between shared genes differentially expressed in *Tbx1*-null and *Pax9*-null datasets. Shared genes (n=329) between the *Tbx1*-null and *Pax9*-null datasets were compared to see if they were concordant (i.e. genes went up in both datasets, or down), or if they were discordant (i.e. went up in one dataset but down in the other). This revealed that 35% of shared genes were both down, but also 63% of shared genes were down in *Tbx1*-nulls and up in *Pax9*-nulls. Examples of genes in each category are shown in the **Table**.

Development • Supplementary information

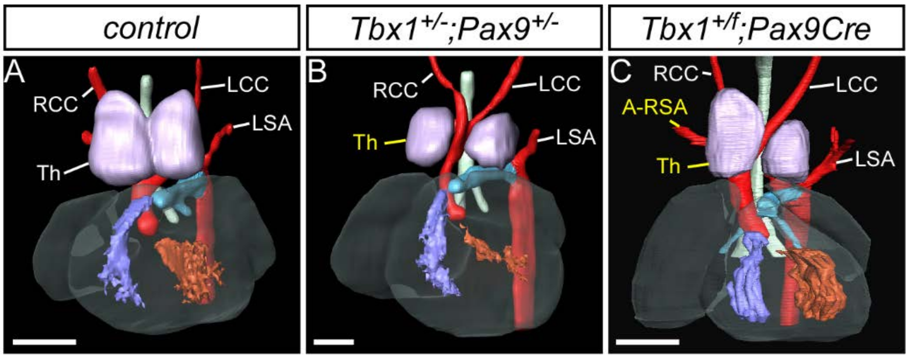

Figure S4. Thymus abnormalities are seen in *Tbx1;Pax9* mutant embryos.
An abnormally placed thymus was frequently observed in conjunction with arch artery defects. **A,** Normally placed thymic lobes (*purple*) in a wild-type control embryo. The thymic lobes are placed ventrally to the aortic arch arteries. **B, C,** In a *Tbx1*$^{+/-}$*;Pax9*$^{+/-}$ mutant embryo (**B**) and a *Tbx1*$^{+/-}$*;Pax9Cre* mutant embryo (**C**), both with interrupted aortic arch and aberrant right subclavian artery (A-RSA), the thymic lobes are split and asymmetrically placed. The right and left common carotid arteries can be seen traversing between the two thymic lobes. Abbreviations: LCC, left common carotid; LSA, left subclavian artery; RCC, right common carotid; Th, thymus. Scale, 500µm.

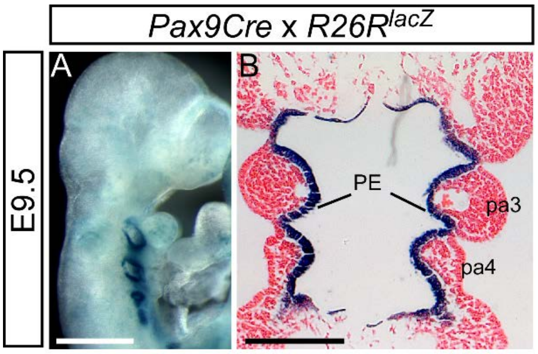

Figure S5. Reporter gene expression from the *Pax9Cre* allele.
A, B, Cre mediated recombination of *lacZ* by *Pax9Cre* induces expression in the pharyngeal endoderm at E9.5. Scale, 500 µm.

Development • Supplementary information

Supplementary Tables

Table S1 (Excel file). Differentially expressed genes ($p \leq 0.1$) from RNA-seq analysis of *Pax9*-null pharyngeal arch tissue. **Sheet A, B,** The top up- and down-regulated coding genes from the *Pax9* RNA-seq data are listed and ranked by log2 fold-change, with a cut off -0.59 for the down-regulated genes (**A**; n=647) and 0.59 for the up-regulated genes (**B**; n=240).
Sheet C, D, Values of the *Tbx1-Pax9* shared genes from the *Pax9* RNA-seq data, ranked by fold-change. **C**, Genes down-regulated in *Pax9*-null pharyngeal arch tissue (n=122). **D,** Genes up-regulated in *Pax9*-null pharyngeal arch tissue (n=220).

Click here to Download Table S1

Table S2. Genotypes of weaned pups (at 3 weeks of age), neonates (collected within 24 h of birth) and embryos (E15.5 and E9.5) from a *Tbx1*$^{+/-}$ and *Pax9*$^{+/-}$ cross. There was a significant loss of expected pups at weaning (Chi-squared test; **** p=1.4x10^{-12}). There was no significant difference from the expected number of pups for each possible genotype in neonates or embryos.

Genotype	Weaning	Neonate	E15.5	E9.5
Wild-type	55	7	16	20
Pax9$^{+/-}$	68	10	15	14
Tbx1$^{+/-}$	43	6	19	17
Tbx1$^{+/-}$;*Pax9*$^{+/-}$	2 ****	9	20	23
Total	*168*	*32*	*70*	*74*
n expected for each genotype	42	8.0	17.5	18.5

Table S3. Summary of thymus phenotypes observed in *Tbx1* and *Tbx1;Pax9* mutant embryos. The thymus was frequently seen to be asymmetric in appearance and split apart in *Tbx1*$^{+/-}$;*Pax9*$^{+/-}$ and *Tbx1*$^{+/flox}$;*Pax9Cre* mutants. An abnormal thymus was always associated with an arch artery defect. One *Tbx1*$^{+/-}$;*Pax9*$^{+/-}$ mutant presented with an absent thymus and an unusual holoprosencephaly type phenotype (not shown).

<table>
<tr><th rowspan="2">Genotype</th><th rowspan="2">Stage</th><th rowspan="2">n</th><th colspan="3">Thymus phenotype</th></tr>
<tr><th>Normal</th><th>Split & asymmetric</th><th>Absent</th></tr>
<tr><td>Tbx1$^{+/-}$</td><td rowspan="2">E15.5</td><td>19</td><td>15 (79%)</td><td>4 (21%)</td><td>0</td></tr>
<tr><td>Tbx1$^{+/-}$;Pax9$^{+/-}$</td><td>20</td><td>3 (15%)</td><td>16 (80%)</td><td>1 (5%)</td></tr>
<tr><td>Tbx1$^{+/flox}$;Pax9Cre</td><td>E13.5 -E15.5</td><td>26</td><td>16 (61.5%)</td><td>10 (38.5%)</td><td>0</td></tr>
</table>

Table S4. Genotypes of neonates (collected within 48 h of birth) from a *Tbx1*$^{+/flox}$ x *Pax9Cre* cross. Foetuses (E13.5 – E15.5) and embryos (E10.5) were from a *Tbx1*$^{flox/flox}$ x *Pax9Cre* cross. There was a significant loss of expected neonates (Chi-squared test; **** p=2.4x10^{-5}).

Genotype	Neonate	Foetus	Embryo
Wild-type	39	-	-
Pax9Cre	43	-	-
Tbx1$^{+/flox}$	33	18	15
Tbx1$^{+/flox}$;*Pax9Cre*	8****	17	17
Total	*123*	*35*	*32*
n expected for each genotype	30.75	17.5	16

Table S5. *PAX9* mutations and aberrant expression levels that have been associated with human disease.

Disease	Mutation/ karyotype	Detail	Other	Reference
Congenital Heart Defect	14q13 (105kb deletion)	IAA-B, BAV, hypoplastic aorta, VSD	Facial dysmorphisms	1
	46,XY,del(14) (11.2q13)	Persistent foramen ovale	Craniofacial defects	2
	46,XY,del(14) (11.2q13)	Patent foramen ovale and patent ductus arteriosus	Craniofacial defects, delayed bone ossification	3
	14q13.3 (884kb deletion)	Persistent pulmonary hypertension of the newborn	Oligodontia, hypothyroidism	4
Craniofacial	*PAX9* mutations	Hypo/oligodontia	-	5,6
		Cleft lip/palate	Hypodontia	7,8
Skeletal	46,XY,t(14;18) (q13;q12)	Mesomelic bone dysplasia	-	9
	Jarcho-Levin Syndrome	Vertebral segmentation defect	Reduction in PAX9 expression	10
Cancer	14q13.3	Lung cancer	PAX9 expression amplified	11

PAX9 is located at 14q13.3.
Abbreviations: BAV, bicuspid aortic valve; IAA-B, interrupted aortic arch type B; VSD, ventricular septal defect.

Development • Supplementary information

Table S6. Penetrance of 4th PAA defects in *Tbx1*$^{+/–}$ and *Tbx1*$^{+/flox}$;*Pax9Cre* mutant embryos assessed from published and our data.

Genotype	Stage	n	% defects [a]	Reference
Tbx1$^{+/–}$	E10.5–E11.0	127	87% (63-100%)	[12-17] & this study
	E11.5	76	62% (50-74%)	[16-18]
	Fetal (E14.5–P2)	215	34% (18-49%)	[12-16,18-20] & this study
Tbx1$^{+/flox}$; *Pax9Cre*	E10.5	17	92%	This study
	Fetal (E13.5–E15.5)	18	33%	This study

[a] For *Tbx1*$^{+/–}$ mice, % defects are given as the mean incidence of abnormalities for the studies cited, with 95% confidence intervals.

Table S7. Penetrance of 4th PAA defects following heterozygous deletion of *Tbx1* (globally or conditionally) from E10.5 embryos and assessed by intra-cardiac ink injection from published and our data.

Genotype	Tissue affected	n	Abnormal	Reference
***Tbx1*$^{+/–}$**	Endoderm, mesoderm, ectoderm	127	87%	[12-17] & this study
***Tbx1*$^{+/–}$; *Pax9*$^{+/–}$**	Endoderm, mesoderm, ectoderm	9	100%	This study
Tbx1*$^{+/flox}$; *Pax9Cre	Endoderm	17	92%	This study
Tbx1*$^{+/flox}$; *Foxg1Cre	Endoderm*	30	0	[21]
Tbx1*$^{+/flox}$; *Mesp1Cre	Mesoderm	19	0	[22]
Tbx1*$^{+/flox}$; *Foxg1Cre	Endoderm, mesoderm, ectoderm	21	42%	[22]
Tbx1*$^{+/flox}$; *Fgf15Cre	Endoderm, ectoderm	34	20%	[22]
Tbx1*$^{+/flox}$; *Hoxa3Cre	Endoderm, mesoderm, ectoderm (neural crest)	17	50%	[22]
Tbx1*$^{+/flox}$; *AP2aCre**	Ectoderm (neural crest)	9	0	[14]

* Mice were maintained congenic in the Swiss Webster background to restrict Cre expression to the pharyngeal endoderm.
** *Tbx1*$^{+/flox}$;*AP2aCre* phenotype assessed at E15.5 by histology.
N.B. *Tbx1* is not expressed in neural crest cells.

Development • Supplementary information

Table S8. Antibodies and probes used for immunostaining and *in situ* hybridisation.

Target	Catalogue number	Species and type	Supplier	Dilution
Primary antibodies				
Pax9	ab28538	Rat monoclonal	Abcam	1:100
Tbx1	ab18530	Rabbit polyclonal		1:100
CD31	ab56299	Rat monoclonal		1:100
GFP	ab6556	Rabbit polyclonal		1:100
ERG	ab92513	Rabbit monoclonal		1:1000
BrDU	ab6326	Rat monoclonal		1:200
Cleaved caspase-3	9661	Rabbit polyclonal	Cell Signalling	1:300
aSMA	a2547	Mouse monoclonal	Sigma	1:200
Secondary antibodies				
Donkey anti-mouse IgG Alexa Fluor 488	A-21202	-	Thermo Fisher Scientific	1:200
Donkey anti-rabbit IgG Alexa Fluor 488	A-21206	-		
Donkey anti-rabbit IgG Alexa Fluor 594	A-21207	-		
Donkey anti-rat IgG Alexa Fluor 488	A-21208	-		
Donkey anti-rat IgG Alexa Fluor 594	A-21209	-		
Goat anti-rat IgG Alexa Fluor 647	A-21247	-		
Nuclear stain				
DAPI	H-1200	-	Vector Laboratories	-
RNAscope probes				
Pax9	454321-C2	Mouse	Advanced Cell Diagnostics	1:50
Tbx1	481911			Direct
Chd7	458031			Direct
Gbx2	314358			1:50

Table S9. qPCR primer sequences

Gene	RefSeq	Primer sequence
Chd7	NM_001081417	GGAGAACCCTGAGTTTGCTG<br>CCCTGAAGTAGAGGCGACAG
eYFP	-	ACGTAAACGGCCACAAGTTC<br>TCGTCCTTGAAGAAGATGGTG
Gapdh	NM_008084	TGTGCAGTGCCAGCCTCGTC<br>TGACCAGGCGCCCAATACGG
Gbx2	NM_010262	GAGGCGGCAACTTCGACAAAGCC<br>TCCTCCTTGCCCTTCGGGTCATC
Pax9	NM_011041	CCGGCACAGACTTCCTTTTA<br>CCTTCCGTTCACGAACACTC
Tbx1	NM_011532	TGAGGAGACACGCTTCACTG<br>CTGCAGCGTCTTTGTCTGAG

References

1. Santen, G.W. *et al.* Further delineation of the phenotype of chromosome 14q13 deletions: (positional) involvement of FOXG1 appears the main determinant of phenotype severity, with no evidence for a holoprosencephaly locus. *J Med Genet* **49**, 366-72 (2012).
2. Schuffenhauer, S. *et al.* De novo deletion (14)(q11.2q13) including PAX9: clinical and molecular findings. *J Med Genet* **36**, 233-236 (1999).
3. Shapira, S.K. *et al.* De novo proximal interstitial deletions of 14q: cytogenetic and molecular investigations. *Am J Med Genet* **52**, 44-50 (1994).
4. Hayashi, S., Yagi, M., Morisaki, I. & Inazawa, J. Identical deletion at 14q13.3 including PAX9 and NKX2-1 in siblings from mosaicism of unaffected parent. *J Hum Genet* (2015).
5. Stockton, D.W., Das, P., Goldenberg, M., D'Souza, R.N. & Patel, P.I. Mutation of PAX9 is associated with oligodontia. *Nature Genetics* **24**, 18-19 (2000).
6. Bonczek, O., Balcar, V.J. & Šerý, O. PAX9 gene mutations and tooth agenesis: A review. *Clinical Genetics* **92**, 467-476 (2017).
7. Das, P. *et al.* Novel missense mutations and a 288-bp exonic insertion in PAX9 in families with autosomal dominant hypodontia. *American Journal of Medical Genetics Part A* **118A**, 35-42 (2003).
8. Ichikawa, E. *et al.* PAX9 and TGFB3 are linked to susceptibility to nonsyndromic cleft lip with or without cleft palate in the Japanese: population-based and family-based candidate gene analyses. *Journal Of Human Genetics* **51**, 38 (2005).
9. Kamnasaran, D. *et al.* Defining the breakpoints of proximal chromosome 14q rearrangements in nine patients using flow-sorted chromosomes. *Am J Med Genet* **102**, 173-82 (2001).
10. Bannykh, S.I. *et al.* Aberrant Pax1 and Pax9 expression in Jarcho-Levin syndrome: Report of two Caucasian siblings and literature review. *American Journal of Medical Genetics Part A* **120A**, 241-246 (2003).
11. Kendall, J. *et al.* Oncogenic cooperation and coamplification of developmental transcription factor genes in lung cancer. *Proceedings of the National Academy of Sciences* **104**, 16663-16668 (2007).
12. Calmont, A. *et al.* Tbx1 controls cardiac neural crest cell migration during arch artery development by regulating Gbx2 expression in the pharyngeal ectoderm. *Development* **136**, 3173-3183 (2009).
13. Guris, D.L., Duester, G., Papaioannou, V.E. & Imamoto, A. Dose-dependent interaction of Tbx1 and Crkl and locally aberrant RA signaling in a model of del22q11 syndrome. *Dev Cell* **10**, 81-92 (2006).
14. Randall, V. *et al.* Great vessel development requires biallelic expression of Chd7 and Tbx1 in pharyngeal ectoderm in mice. *J Clin Invest* **119**, 3301-10 (2009).
15. Vitelli, F., Morishima, M., Taddei, I., Lindsay, E.A. & Baldini, A. Tbx1 mutation causes multiple cardiovascular defects and disrupts neural crest and cranial nerve migratory pathways. *Hum Mol Genet* **11**, 915-22 (2002).
16. Ryckebusch, L. *et al.* Decreased Levels of Embryonic Retinoic Acid Synthesis Accelerate Recovery From Arterial Growth Delay in a Mouse Model of DiGeorge Syndrome. *Circ Res* **106**, 686-694 (2010).
17. Lindsay, E.A. & Baldini, A. Recovery from arterial growth delay reduces penetrance of cardiovascular defects in mice deleted for the DiGeorge syndrome region. *Hum Mol Genet* **10**, 997-1002 (2001).
18. Papangeli, I. & Scambler, P.J. Tbx1 genetically interacts with the transforming growth factor-beta/bone morphogenetic protein inhibitor Smad7 during great vessel remodeling. *Circ Res* **112**, 90-102 (2013).

Development • Supplementary information

19. Zhang, Z. & Baldini, A. In vivo response to high-resolution variation of Tbx1 mRNA dosage. *Hum Mol Genet* **17**, 150-7 (2008).
20. Aggarwal, V.S. *et al.* Dissection of Tbx1 and Fgf interactions in mouse models of 22q11DS suggests functional redundancy. *Hum Mol Genet* **15**, 3219-28 (2006).
21. Arnold, J.S. *et al.* Inactivation of Tbx1 in the pharyngeal endoderm results in 22q11DS malformations. *Development* **133**, 977-87 (2006).
22. Zhang, Z. *et al.* Tbx1 expression in pharyngeal epithelia is necessary for pharyngeal arch artery development. *Development* **132**, 5307-15 (2005).

Development • Supplementary information
